# Inhibition of casein kinase 1 δ/ε improves cognitive performance in adult C57BL/6J mice

**DOI:** 10.1038/s41598-021-83957-9

**Published:** 2021-02-26

**Authors:** Heather Mahoney, Emily Peterson, Hannah Justin, David Gonzalez, Christopher Cardona, Korey Stevanovic, John Faulkner, Amara Yunus, Alexandra Portugues, Amy Henriksen, Camden Burns, Cameron McNeill, Joshua Gamsby, Danielle Gulick

**Affiliations:** 1 Byrd Alzheimer’s Institute, University of South Florida Health, Tampa, FL USA; 2 Department of Molecular Medicine, Morsani College of Medicine, University of South Florida, Tampa, FL USA; 3 National Institute of Environmental Health Sciences, National Institute of Health, Research Triangle Park, NC USA; 4 College of Pharmacy, University of Florida, Gainesville, FL USA; 5 USF Health Informatics Institute, University of South Florida Health, Tampa, FL USA

**Keywords:** Circadian rhythms and sleep, Cognitive neuroscience

## Abstract

Time-of-day effects have been noted in a wide variety of cognitive behavioral tests, and perturbation of the circadian system, either at the level of the master clock in the SCN or downstream, impairs hippocampus-dependent learning and memory. A number of kinases, including the serine-threonine casein kinase 1 (CK1) isoforms CK1δ/ε, regulate the timing of the circadian period through post-translational modification of clock proteins. Modulation of these circadian kinases presents a novel treatment direction for cognitive deficits through circadian modulation. Here, we tested the potential for PF-670462, a small molecule inhibitor of CK1δ/ε, to improve cognitive performance in C57BL/6J mice in an array of behavioral tests. Compared to vehicle-treated mice tested at the same time of the circadian day, mice treated with PF-670462 displayed better recall of contextual fear conditioning, made fewer working memory errors in the radial arm water maze, and trained more efficiently in the Morris Water Maze. These benefits were accompanied by increased expression of activity-regulated cytoskeleton-associated protein (Arc) in the amygdala in response to an acute learning paradigm. Our results suggest the potential utility of CK1δ/ε inhibition in improving time-of-day cognitive performance.

## Introduction

Over the past two decades, the complex processes that underlie cognition have been elucidated. These processes are profoundly affected by changes in innate circadian timekeeping mechanisms^1^, with time-of-day effects on cognitive performance persisting even in the absence of external timing cues^2–5^. While disruption of daily sleep–wake patterns and cognitive impairment frequently manifest in neurological disorders like Alzheimer’s disease^6,7^, circadian rhythm disruptions also occur in otherwise healthy individuals when the endogenous circadian clock cannot adapt to a voluntary or involuntary shift in the timing of sleep^8^. The structure of modern society imposes cognitive demands at all times of day, including those times at which we are not primed for peak cognitive performance. This is common during jet lag, shift work, and many school schedules, and is exacerbated by the sharp increase in the use of digital devices at night and consequent exposure to wake-promoting blue light^9–12^. Alterations to clock timing are associated with cognitive deficits^1^, and increasing evidence suggests that circadian rhythm disruption contributes to a number of other health issues^13^. Circadian disruption is usually addressed through chronotherapy—interventions to correct the mismatch between exogenous time and endogenous sleep timing in order to improve the ability to achieve and maintain sleep^14^. Chronotherapeutics include behavioral therapies, such as strategic exposure to specific wavelengths of light to realign the endogenous clock^15^. Melatonin, benzodiazepines, and caffeine have also been effective in short-term jet-lag conditions, but produce undesirable side effects and have limited efficacy for long term circadian disruption in shift workers^16^. In the current study, we investigate the use of a pharmacological intervention that acts on circadian clock mechanisms to improve cognition in healthy adult mice, potentially by altering the timing of the internal circadian clock, which would shift the peaks and troughs of cognitive capacity.

The circadian clock is an endogenous system that controls the rhythmicity of physiological and biochemical processes, thus enabling organisms to anticipate daily changes in the environment. The master circadian pacemaker resides in the suprachiasmatic nucleus (SCN) of the hypothalamus. The SCN conveys time-of-day information to peripheral oscillators, including the hippocampus and amygdala, essential memory processing areas^17–19^. This master circadian oscillator can be reset or “entrained” by exogenous cues called zeitgebers, which allow the system to adapt to changes in the environment and keep circadian rhythms aligned to their appropriate phase. At the molecular level, the mammalian molecular clock is present in all nucleated cells and consists of internal, interlocked positive and negative transcription-translation feedback loops^17^. At the core of the negative feedback loop are the heterodimeric Brain and Muscle ARNT-Like (BMAL) and Circadian Locomotor Output Cycles Kaput (CLOCK) transcription factors, which drive the expression of the negative regulator *Period* (*Per*) and *Cryptochrome* (*Cry*) gene families^17,20^. Once transcribed, PER and CRY are ultimately translocated to the nucleus to repress their own transcription until their proteasome-mediated degradation releases the molecular “brake” of the clock, allowing BMAL-CLOCK transcription to resume and resetting the 24 h period^21,22^. A number of kinases, including the serine-threonine casein kinase 1 (CK1) isoforms CK1ε and CK1δ, modulate the timing of the circadian period through post-translational modification of clock proteins^23,24^.

Many studies have demonstrated time-of-day effects in a wide variety of cognitive behavioral tests, across many different animal models (reviewed by Smarr et al.^25^). Perturbation of the SCN or circadian rhythms impairs hippocampus-dependent learning and memory. While the SCN acts as the central pacemaker, a cell-autonomous clock has also been identified in the hippocampus, driven by the SCN but capable of oscillating in isolation^26,27^. The hippocampus is essential for most forms of declarative learning and memory, and hippocampal cells rhythmically express clock genes even in the absence of input from the SCN^19,28,29^. In fact, PER2 is highly expressed in hippocampal pyramidal cells, and the rhythm of its expression is nearly 180° out of phase with SCN expression patterns^28,30^. Mice with mutations in PER1 or PER2 have deficits in long-term memory, and PER2-deficient mice exhibit abnormal long-term potentiation and reduced phosphorylation of cAMP-response-element-binding protein (CREB) in the hippocampus^19,28^.

CK1δ inhibition increases nuclear retention of PER2, lengthens the circadian period, and stabilizes disrupted circadian rhythms in arrhythmic genetic and environmental models^31^. Our previous work demonstrates the ability of CK1δ/ε inhibition with the small-molecule inhibitor PF-670462 to improve cognitive performance and reduce amyloid burden in a mouse model of Alzheimer’s disease^32^. To expand our understanding of the effects of CK1 inhibition on learning and memory systems, we examined changes in cognitive behaviors in wild-type (WT) C57BL/6J mice during chronic, short-term treatment with PF-670462. Based on evidence that CK1δ is the dominant regulator of circadian period length, and that inhibition of CK1δ stabilizes circadian rhythms in mouse models of disrupted circadian timing, we chose to chronically treat adult WT mice with 10 mg/kg/day PF-670462 to preferentially inhibit CK1δ over CK1ε^31,33^. To determine whether CK1δ inhibition can improve behavioral performance at a time of day in which mice display poorer cognitive performance^1^, we subjected mice to a battery of behavioral tests during their normal rest phase in the light cycle of a 12/12 light:dark (LD) cycle, including cognitive, affective, and motor tests. We hypothesized that daily treatment with a CK1δ inhibitor may increase performance on these tasks compared to vehicle-treated mice tested at the same time, as the PF-670462-treated mice might no longer be in a trough of cognitive capacity due to the phase-shifting effects of the drug. As PF-670462 has also been shown to stabilize disrupted circadian rhythms, we expected that this effect might be exaggerated in multi-day tasks that disrupt sleep–wake cycles over several days. To generate a snapshot of the expression of key circadian and learning and memory proteins at the time of testing, we collected tissue and examined BMAL1, PER1, PER2, phosphorylated extracellular signal-regulated kinase (pERK), and glycogen synthase kinase 3 beta (GSK3β) expression in the hippocampus 24 h after the end of behavioral testing. Finally, we presented a separate cohort of mice with an acute learning experience and assessed the effects of CK1δ inhibition on the activation of activity-regulated cytoskeleton-associated protein (Arc), an immediate early gene associated with memory, in the hippocampus and amygdala^34^. We find that selective inhibition of CK1δ produces a mild improvement in the cognitive performance of WT mice that is associated with an alteration in the expression of learning and memory-related genes in the hippocampus and amygdala.

## Results

To determine whether the cognitive performance of healthy, adult WT mice could be improved by chronic inhibition of CK1δ with PF-670462, we treated 2–4 month old male C57BL/6J mice with PF-670462 (CK1i, 10 mg/kg/day) or vehicle (VEH, 20% b-cyclodextrin in citrate buffer) at ZT10 (2 h before lights-off) for 3 days, then began a battery of behavioral tests and continued to administer VEH or CK1i at ZT10 daily throughout testing.

While inhibition of CK1ε has a minimal effect on period length, selective inhibition of CK1δ lengthens the period of behavioral rhythms^31,35^. For this reason, we focused our investigation on CK1δ inhibition, hypothesizing that cognitive performance rhythms and time-of-day gating of learning and memory in the hippocampus may also be altered. We selected our dosing based on Meng et al.^31^, using a dose of PF-670462 (10 mg/kg) that is expected to produce a drug concentration in the brain of approximately threefold IC_50_ for CK1δ and 0.7-fold for CK1ε^33^. Our experimental conditions were modeled after several studies that administered PF-670462 at or near ZT10 to correspond with peak CK1δ/ε activity in the SCN in order to generate the maximum effect on the circadian phase^31,33,36^.

**Figure 1:**
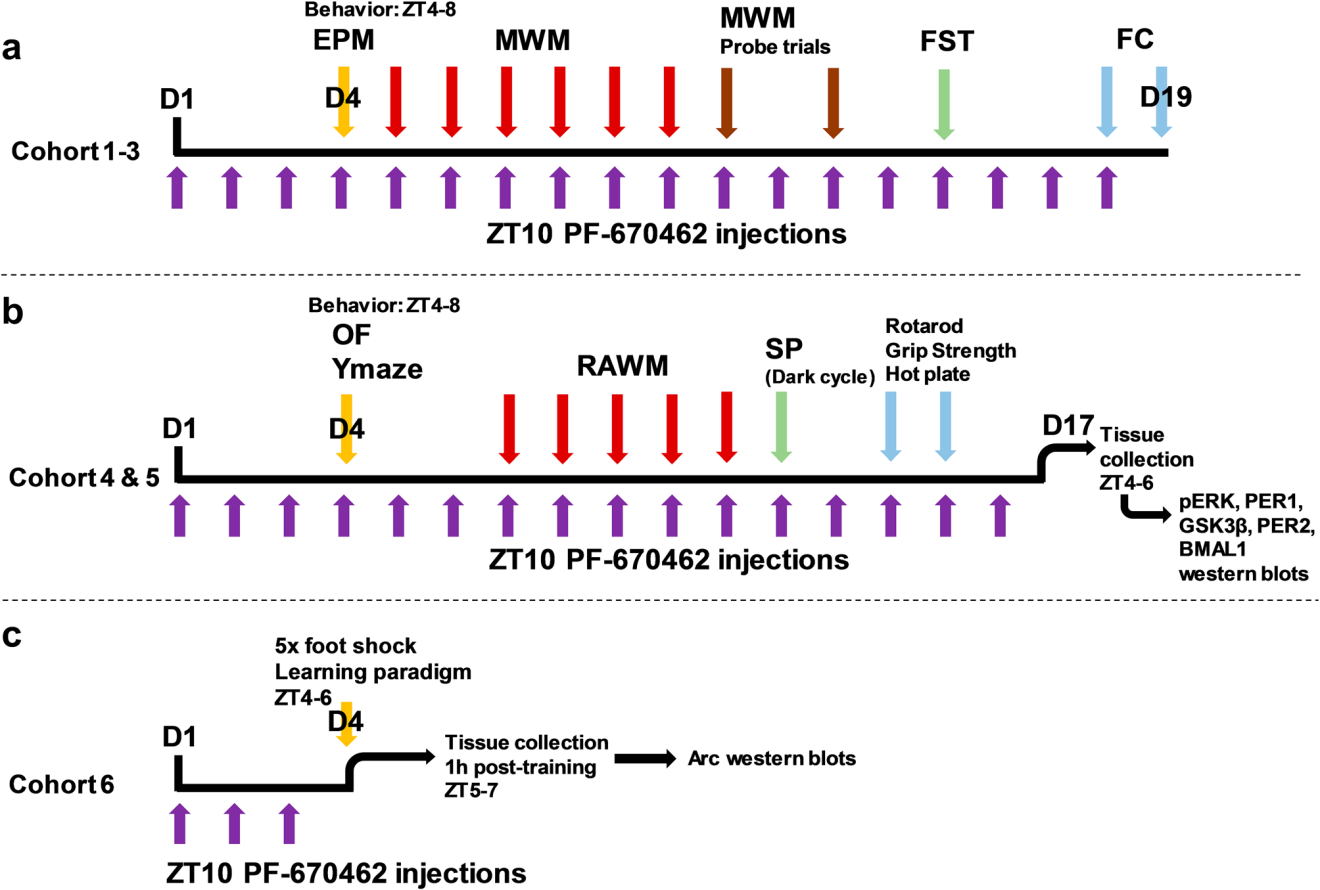
Experimental design. (**a**) Cohort 1–3 schedule, consisting of 18 consecutive days of ZT10 CK1i injections, and elevated plus maze (EPM), Morris water maze (MWM), Porsolt forced swim test (FST), and fear conditioning (FC). (**b**) Cohort 4 and 5 schedule, consisting of 16 consecutive days of ZT10 CK1i injections, open field (OF), Ymaze, 6-arm radial arm water maze (RAWM), sucrose preference test (SP), Rotarod, grip strength, and hot plate tests followed by tissue collection. (**c**) Arc experiment schedule, consisting of 3 days of ZT10 CK1i injections followed by a 5-shock fear conditioning learning paradigm, and tissue collection 1 h post-training.

Whenever possible, our schedule of testing was designed to place the most stressful tests at the end of the testing period, to avoid stress effects on cognitive behaviors. Because water maze testing is also a stressor, we performed a sucrose preference test immediately following radial arm water maze (RAWM) to detect stress-induced anhedonia. To minimize over-time effects on our results and test primarily between ZT4-8, we split testing into multiple cohorts. (Fig. 1a,b).

### Chronic, short-term treatment with PF-670462 causes no obvious adverse effects

**Figure 2:**
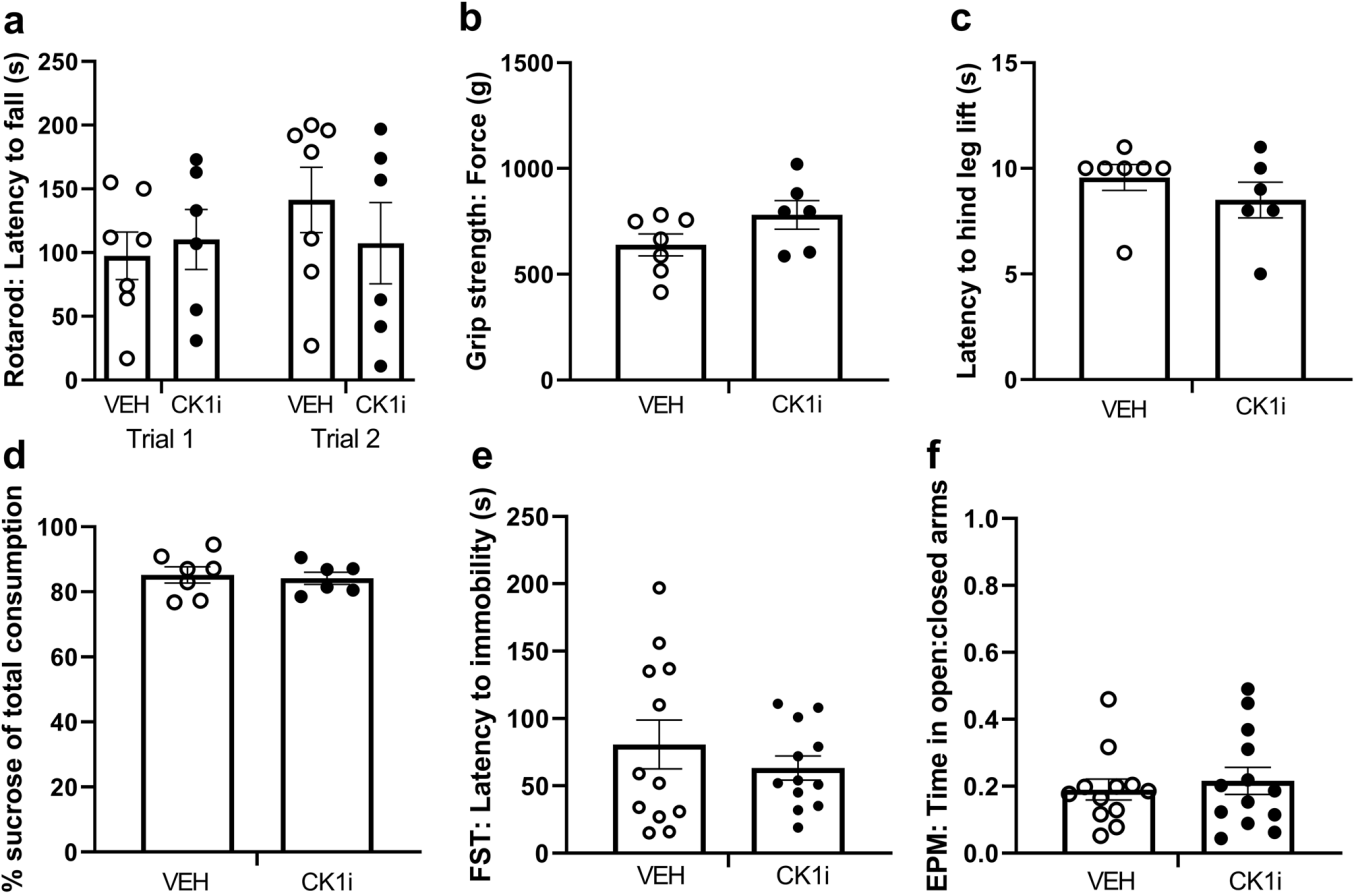
Chronic, short-term treatment with PF-670462 causes no obvious behavioral deficits. (**a**) Latency to fall from Rotarod over two trials (VEH: n = 7 mice, CK1i: n = 6 mice). (**b**) Forelimb grip strength, presented as the average of three trials per mouse. (VEH: n = 7 mice, CK1i: n = 6 mice). (**c**) Latency (s) to hind leg lift on hot plate. (VEH: n = 7 mice, CK1i: n = 6 mice). (**d**) Percent preference for a 2% sucrose solution over water in a two bottle, overnight test. (VEH: n = 7 mice, CK1i: n = 6 mice). (**e**) Latency (s) to first bout of immobility in the forced swim test (VEH: n = 12 mice, CK1i: n = 13 mice). (**f**) Ratio of time in the open:closed arms of the elevated plus maze. (VEH: n = 12 mice CK1i: n = 13 mice) Each dot represents a mouse. Data are represented as mean ± SEM.

We included several assays to determine whether chronic, short-term treatment with CK1i would alter behaviors that might confound the results of our cognitive tests. To test for intact locomotor activity and normal forelimb strength, which are essential for performance of the water maze tasks, we used a two-trial rotarod test and measured forelimb grip strength. We found no differences in locomotor ability on the rotarod (F(1,11) = 0.9777, p = 0.344) (Fig. 2a), or in grip strength (t(11) = 1.687, p = 0.1198) (Fig. 2b). To ensure intact nociception and to rule out stress-induced anhedonia, we confirmed that there were no differences in hot plate (t(11) = 1.047, p = 0.3175) (Fig. 2c), or sucrose preference tests(t(11) = 0.3228, p = 0.7529) (Fig. 2d). We used the Porsolt forced swim test (FST) and elevated plus maze (EPM) to look for differences in affect and anxiety. We found no differences in latency to immobility in the FST (t(22) = 0.8655, p = 0.3961) (Fig. 2e), or in the ratio of time spent in the open vs. closed arms in the EPM (t(23) = 0.5056, p = 0.6179) (Fig. 2f).

### PF-670462 improves contextual fear conditioning

**Figure 3:**
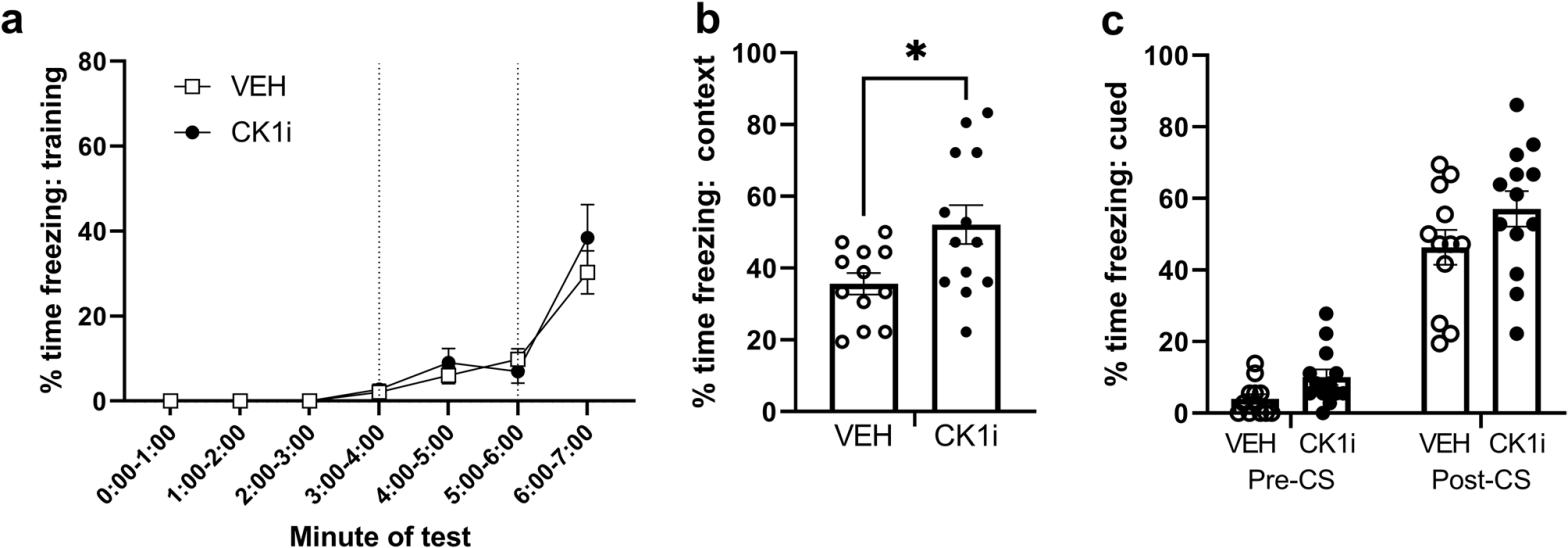
Mice treated with 10 mg/kg PF-670462 display increased freezing behavior in contextual fear conditioning, but have normal training behavior and performance on the cued test. (**a**) Freezing behavior over time during training. Dotted lines indicate presentation of tone-shock pairing. (VEH: n = 12 mice, CK1i: n = 13 mice). (**b**) Freezing behavior in the contextual test (VEH: n = 12 mice, CK1i: n = 13 mice). (**c**) Freezing behavior in the cued test (VEH: n = 12 mice, CK1i: n = 13 mice). Each dot represents a mouse. Data are represented as mean ± SEM. *p < 0.05, Student's t-test with Welch’s correction.

To determine whether it is possible to use CK1δ inhibition to improve performance on learning and memory tasks during a time of off-peak cognitive efficiency, we tested during the light cycle and used several different behavioral tests for which day/night differences in peak/nadir performance have been demonstrated in prior studies^1^. Our first set of experiments included classical fear conditioning (FC) with both contextual and cued retrieval tests. In FC training, both VEH and CK1i-treated mice increased their freezing behavior following the two tone-shock pairings. A repeated measures ANOVA demonstrated a significant effect of time (F(1.507, 33.15) = 39.86, p < 0.0001), but not treatment (p = 0.5626, F(1, 23) = 0.3451), and no time x treatment interaction (F(6, 132) = 0.7735, p = 0.5921) (Fig. 3a). In the context test, the total percent time freezing was higher in CK1i-treated mice (t(18.63) = 2.658, p = 0.0157) (Fig. 3b), while there was no difference in the cued test, either before (t(23) = 2.328, p = 0.0573) or after (t(23) = 1.543, p = 0.1365) the presentation of the conditioned stimulus. (Fig. 3c).

### CK1 inhibition with PF-670462 improves spatial learning

**Figure 4:**
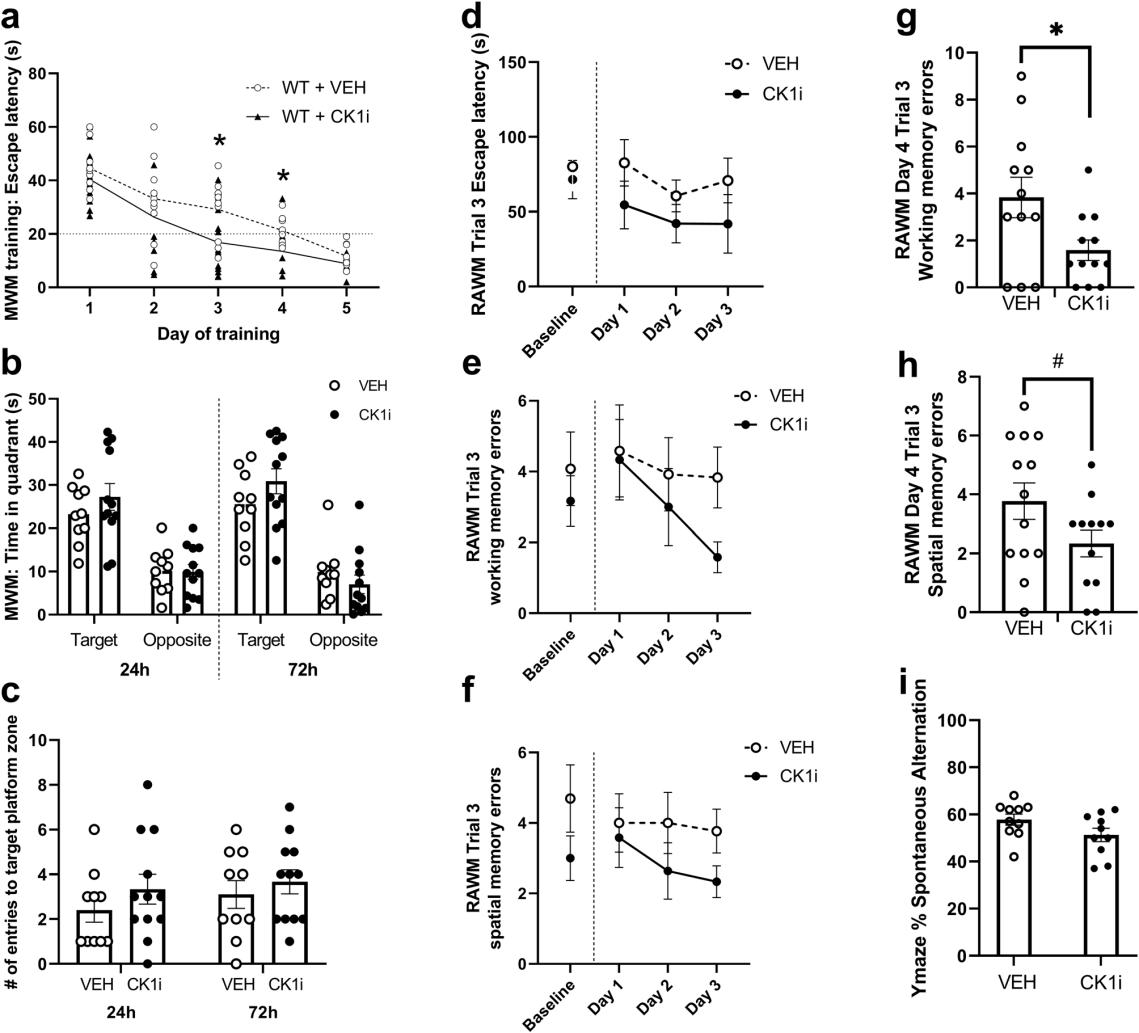
CK1 inhibition with PF-670462 improves spatial learning behavior but not recall in the Morris water maze, and working memory performance in the radial arm water maze but not Y maze. (**a**) Escape latency over 5 days of training in the Morris water maze (MWM). Dotted line indicates goal latency of 20 s. (**b**) Time spent in target and opposite quadrants of the MWM pool during probe trials conducted 24 and 72 h after the completion of training. (**c**) Number of entries during the probe trials to the area of the pool that previously contained the escape platform. (**d**) Escape latency from the 6-arm radial arm water maze (RAWM) over 4 days of testing. (**e**) Working memory type errors over time in the RAWM. (**f**) Spatial memory type errors over time in the RAWM. (**g**) Working memory type errors in RAWM in the final trial on the last day of testing. (**h**) Spatial memory type errors in RAWM in the final trial on the last day of testing. (**i**) Percent spontaneous alternation in the Y-maze. Each dot represents a mouse. Data are represented as mean ± SEM. *p < 0.05, ^#^p = 0.078.

In our first set of behavioral tests, we also used Morris water maze (MWM) to measure spatial learning and long-term memory retrieval. In the MWM, both groups trained successfully to an escape latency of less than 20 s by day 5 of training. A repeated measures ANOVA was significant for training day (F(2.414, 45.88) = 31.67, p < 0.0001) and treatment (F(1, 20) = 9.365, p = 0.0062), but not the interaction (F(4, 76) = 0.7439, p = 0.5650). CK1i-treated mice found the escape platform more quickly on the third (t(20) = 2.4363, p = 0.0243) and fourth (t(19) = 2.5911, p = 0.0179) days of training. By day 5 of training, untreated mice had similar escape latency to CK1i-treated mice (t(19) = 1.8451, p = 0.0807) (Fig. 4a). We found no difference in the amount of time spent in the target or opposite quadrant, or in the number of entries to the target platform area in either the 24 or 72 h probe tests. (Fig. 4b,c).

Although the results from FC and MWM in our first set of behavioral tests both suggested mild improvement in cognitive performance, we found a low effect size and wanted to determine whether there were effects on other measures of hippocampus-dependent working memory. We used radial arm water maze (RAWM) to examine both spatial and working memory, and Y-maze to examine working memory in a different context. While group differences over time for the 3 training days were not significant (Fig. 4d–f), we found that CK1i-treated mice made fewer working memory errors in the third trial of the final testing day (t(16.27) = 2.335, p = 0.0327) (Fig. 4g). There was also a trend toward fewer spatial memory errors in CK1i-treated mice on this trial (t(23) = 1.845, p = 0.078) (Fig. 4h).

Despite making fewer working memory-type errors in the RAWM, CK1i-treated mice made a similar number of spontaneous alternations as VEH-treated mice in the Y-maze (t(18) = 1.754, p = 0.097) (Fig. 4i), and made a similar number of total arm entries (t(19) = 1.011, p = 0.325). The difference in groups comparisons between RAWM and Y-maze may be due to differences between the tests: Y-maze relies on natural alternating behavior to reflect working memory, while RAWM is dependent on the navigational memory over the course of several days with the added factor of motivation to escape.

### CK1 inhibition with PF-670462 may alter expression of pERK in the hippocampus

**Figure 5:**
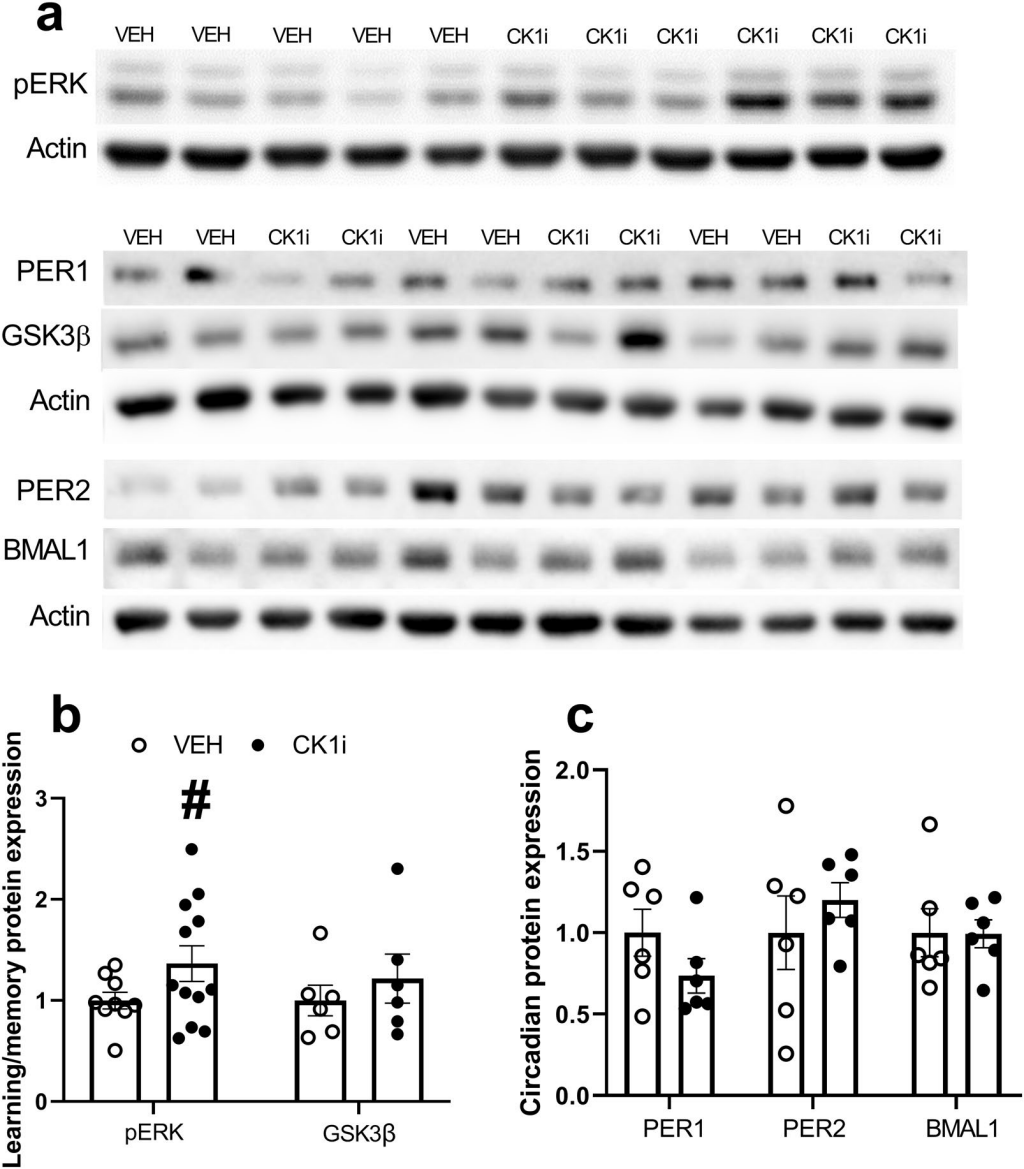
CK1 inhibition may increase pERK expression in the hippocampus. (**a**) Western blots for hippocampal pERK, PER1, GSK3β, PER2, BMAL1, and corresponding β-actin. Representative blot images. Images have been cropped. Full length blot images in Supplementary Fig. S1A–F. Quantification of western blots for (**b**) pERK and GSK3β. pERK results represent two separate normalized gels. (**c**) PER1, PER2, and BMAL1. Bands were normalized to actin, and expressed as fold change from vehicle group mean. Each dot represents result from one mouse. Data are represented as mean ± SEM. ^#^p = 0.079.

To uncover what mechanisms might underlie the improvements in performance that we saw in some of the cognitive tests, we collected hippocampi from test mice and performed western blotting to detect changes in the expression of circadian and learning and memory associated proteins (Fig. 5a). We euthanized the mice as close as possible to ZT6, the approximate midpoint time that we performed our behavioral testing, and alternated groups to help prevent time-of-day effects. We found a trend toward increased phosphorylated ERK, however, the results were variable and a Welch’s t-test showed that this difference was not statistically significant (t(15.369) = 3.552, p = 0.079) (Fig. 5b).

We also examined the “snapshot” expression of several circadian proteins in the hippocampus during the time of behavioral testing to determine whether our behavioral results could be explained by differences in the molecular circadian clock in the hippocampus. We found no significant differences in the expression of PER1 (t(10) = 1.484, p = 0.169), PER2 (t(10) =  − 0.806, p = 0.439), or BMAL1 (t(10) = 0.042, p = 0.967) (Fig. 5c).

### Expression of Arc is increased in the right amygdala of CK1i-treated mice following a negatively-valenced learning event

**Figure 6:**
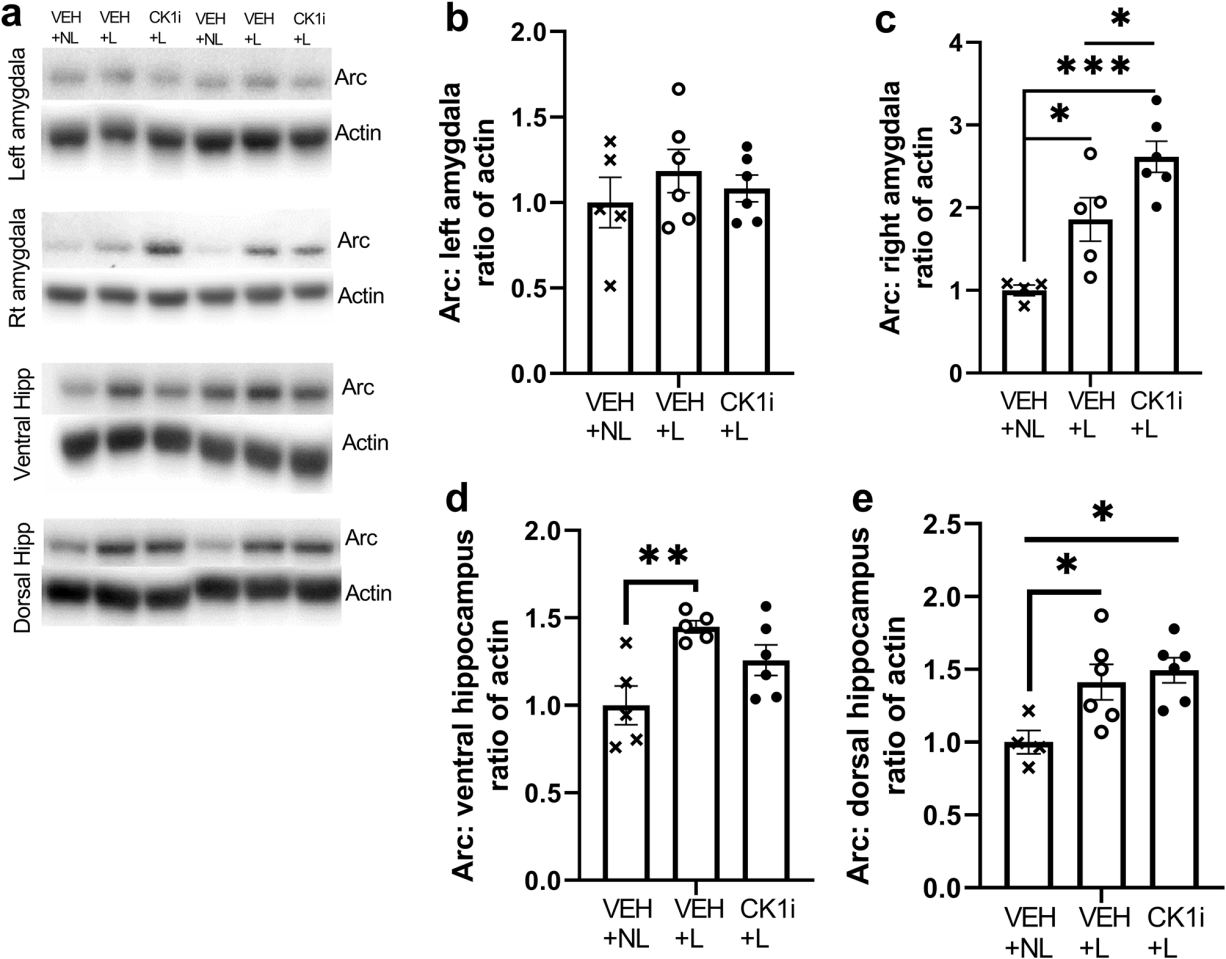
CK1i treatment increases arc activation in the right amygdala. (**a**) Western blots for Arc and actin for mice in three groups: VEH treated and kept in home cage (VEH + NL), VEH treated and exposed to an acute learning paradigm of 5 foot shocks in the fear conditioning chamber (VEH + L), and CK1i treated and exposed to the acute learning paradigm (CK1i + L). Representative blot images. Images have been cropped. Full length blot images in Supplementary Fig. S1G,H. Quantification of western blot results for Arc in the (**b**) left amygdala, (**c**) right amygdala, (**d**) ventral hippocampus and (**e**) dorsal hippocampus of mice in. Bands were normalized to actin, and expressed at fold change from vehicle group mean. Data are represented as mean ± SEM. *p < 0.05, **p < 0.01, ***p < 0.001.

To further investigate potential mechanisms for the improvement in behavioral performance, we used an acute, 5 shock-tone pairing learning paradigm with a separate cohort of mice and collected tissue precisely 1 h after the end of conditioning (Fig. 1c). This design allowed us to examine the expression of Arc, which is transcribed following the release of norepinephrine in response to learning and is essential for hippocampal long-term potentiation and aversive memory formation in the amygdala^37,38^ (Fig. 6a). Previous studies have demonstrated that Arc expression is preferentially upregulated in the right amygdala following a negatively-valenced emotional learning event^34^. A one-way ANOVA indicated no significant differences in left amygdala Arc expression between VEH-treated mice maintained in the home cage with no learning event (VEH + NL), VEH-treated mice exposed to the learning paradigm(VEH + L), and CK1i-treated mice exposed to the learning paradigm (CK1i + L) (F(2,12) = 0.4719, p = 0.6349) (Fig. 6b). However, we found significant group differences in right amygdala Arc expression (CK1i + L) (F(2,12) = 15.18, p = 0.0005); right amygdala Arc was significantly upregulated in both VEH + L (p = 0.0391) and CK1i + L(p = 0.0004) groups compared to VEH + NL. Arc expression in the CK1i + L group was also significantly higher than in the VEH + L group (p = 0.0436) (Fig. 6c). We also found that the learning paradigm altered Arc expression in the dorsal (F(2,12) = 5.5166, p = 0.0184) and ventral (F(2,13) = 6.669447, p = 0.0102) hippocampus. While the learning paradigm increased Arc expression in the VEH group in both the dorsal (p = 0.0183) and ventral (p = 0.0079) hippocampus, it was only increased in the CK1i + L group in the dorsal hippocampus (p = 0.0183), and we found no differences between VEH + L and CK1i + L treatment groups in either. Interestingly, Arc expression in the CK1i + L group was not significantly increased from the aVEH + NL group in the ventral hippocampus (**d**,**e**).

## Discussion

The overexpression of CK1δ/ε in the human Alzheimer’s disease brain has driven research interest in targeting CK1δ/ε for therapeutic intervention, and studies in multiple animal models of AD have demonstrated the ability of CK1δ/ε inhibitors to stabilize circadian rhythms, improve cognitive function, and rescue hippocampal proteomic alterations^39–41^. We have previously shown that inhibition of CK1δ/ε with PF-670462 improves cognitive-affective behavior in the APP-PS1 transgenic mouse model of AD^41^. With this study, we sought to expand our understanding of how inhibition of these key circadian regulators might interact with learning and memory systems in the absence of pathological deficits.

CK1δ/ε inhibition with PF-670462 lengthens the circadian period in cyclic 12:12 LD lighting, despite expectations that light would act as the dominant zeitgeber in these conditions^42^. The ability of CK1δ/ε inhibition to overwhelm light as the primary zeitgeber suggests its potential utility to address circadian dysfunction by modulating oscillations. Studies demonstrating the ability of CK1δ/ε inhibitors to alter peripheral clock gene rhythms have led to the proposal that drugs might be developed to more efficiently phase-adjust circadian rhythms to ease the effect of shift work or jet lag on metabolic processes^43^. Here, we asked whether CK1δ/ε inhibition might also be used to improve cognitive performance in the absence of disease, potentially by shifting the phase of the internal circadian clock to a time of greater cognitive capacity. The oscillation of both clock genes and learning and memory-related genes in the hippocampus suggests that these rhythms might be targetable^1,44^.

Learning and memory efficiency is gated over the day-night cycle^4,5,45^, and these diurnally regulated effects on cognition persist even in the absence of light cues or other external zeitgebers^1,3^. Our molecular results suggest that CK1δ/ε inhibition may interact with learning and memory systems in the hippocampus and amygdala to improve light cycle performance on behavioral tasks dependent on these areas of the brain, even in the absence of major circadian disruption. One likely mechanism for this is a shift in the time-of-day efficiency of these processes resulting from the period-lengthening effects of CK1δ inhibition. We showed improved learning and memory performance in CK1i-treated mice compared to VEH-treated mice tested at the same time of day. Treatment with PF670462 has been shown to lengthen circadian rhythms, so these improvements can potentially be attributed to these groups being tested in different phases—corresponding to differences in the efficiency of learning and memory processes. We chose a testing time in the light cycle, during which a nocturnal animal might be expected to perform poorly on these tasks, to best reveal these differences, if present. However, there is some disagreement in the results of previous studies on the rhythms of acquisition and recall in fear conditioning, and some studies on C57Bl/6 mice with methodology most similar to ours show a nadir of performance at night, and a peak around ZT4^3,5,46^. Rodent water maze studies are also somewhat inconsistent across species and methodology^1^. Because we found mild improvements in several behavioral tests with different peak performance times, another intriguing possibility is that treatment with PF-670462 is interacting with learning and memory mechanisms in some other way. For example, CK1i treatment could have a stabilizing effect on the mild circadian disruption caused by behavioral testing undertaken during the rodents’ sleep cycle, as sleep is a potent modulator of learning and memory.

While the effects of CK1δ/ε inhibition on circadian rhythms make a phase shift to a time of greater cognitive capacity the simplest explanation for our results, CK1 isoforms are widely distributed and have a number of other functions in the brain. All major CK1 isoforms are expressed in the cortex and striatum, and negatively regulate NMDA receptor-mediated glutamatergic synaptic transmission in the cortex and striatum^47^. While multiple studies have shown that CK1δ/ε are highly expressed in the hippocampus, to our knowledge there are no studies to date examining the function of CK1 isoforms on synaptic transmission in the hippocampus^48^. Hippocampal learning and memory is initiated by NMDA-mediated calcium influx and the downstream activation of multiple signaling pathways, including the ERK and mitogen-activated protein kinase (MAP) pathways^49–51^. Our results show a mild increase in pERK in the hippocampus of mice treated with PF-670462, though this difference failed to reach significance. It is unclear whether this increase is due to alteration of the circadian phase of rhythmically expressed kinases in the hippocampus, or perhaps by more direct action of CK1δ/ε inhibition on the regulation of synaptic transmission. We found no differences in hippocampal expression of core circadian proteins PER1, PER2, or BMAL1, which might suggest that CK1δ/ε with PF-670462 has minimal effect on the molecular circadian clock in the hippocampus. Time-course studies of circadian and learning and memory protein expression in the hippocampus will be essential to uncovering the mechanisms behind the cognitive effects of CK1δ/ε inhibition.

Another possible explanation for the cognitive improvements with PF-670462 treatment is direct action on learning and memory pathways. If CK1δ, an abundant serine-threonine kinase, phosphorylates proteins involved in learning and memory cascades, its inhibition could lead to downstream effects on behavior. For example, GSK3β is a recognized regulator of hippocampal function and synaptic plasticity with an endogenous circadian rhythm in phosphorylation state in area CA1 of the hippocampus^52^. While we found no significant change in the expression of GSK3β in the hippocampi of our CK1i-treated mice, this does not rule out changes in inhibitory phosphorylation that may underlie differences in day-night cognitive performance^53^. While we consider these results in the context of previous work demonstrating the inhibitory potential of PF-670462 toward CK1δ/ε, kinase specificity assays indicate that it may also inhibit other CK1 isoforms including CK1α^54^. The CK1 isoforms have a wide variety of biological roles^55^, including modulation of circadian rhythms, and the specificity of inhibition with PF-670462 must be considered in future work that attempts to determine the mechanisms the improvements in cognition we show.

The most promising evidence for a molecular correlate to the behavioral results we see in this study is in the amygdala. We found an increase in ARC protein expression in the right amygdala of CK1i-treated mice exposed to an acute, negative learning experience. Previous studies show a critical role for ARC expression in synaptic plasticity and memory consolidation, lateralized to the right amygdala for negatively-valenced learning events^34,56,57^. Our behavioral results show increased freezing with PF-670462 treatment in contextual but not cued fear conditioning, and while the hippocampus is essential for learning and memory, the amygdala also plays a role in both types of fear-based associative learning. It seems likely that CK1δ/ε inhibition, or its circadian sequelae, have effects on multiple brain areas and signaling pathways.

Our results reflect behavioral differences at a single timepoint, which is an important limitation of this study. To determine the precise mechanisms for how CK1δ/ε inhibition improves cognitive performance, future studies at multiple timepoints will be necessary to determine whether the cognitive improvement is time-of-day dependent, or remains regardless of testing time. If the former proves true, these effects are likely due, at least in part, to a treatment-driven phase shift altering the timing of the rhythms of cognitive capacity. Regardless, our results suggest that treatment with PF-670462 or other forms of chronotherapy may be effective not only as a treatment for cognitive symptoms and circadian disruption in AD, but also in treating more subtle circadian-related cognitive impairment. These results reflect the interactions between circadian and learning and memory systems, and most interestingly, demonstrate cognitive enhancement in normal, healthy adult mice which may be of significant translational value. Future studies should investigate the mechanisms underlying this cognitive enhancement and focus on CK1δ/ε inhibition as a tool to better understand rhythmicity and circadian control of learning and memory systems.

## Methods

### Mice

We used male C57BL/6J WT mice from Jackson laboratory, aged 2–3 months at the beginning of drug administration and behavioral testing. Studies we restricted to male mice to reduce the number of mice necessary to complete the study while avoiding potentially confounding effects of the estrous cycle. Mice were group housed in 12 h LD in standard cages and fed standard mouse chow and water ad libitum*.* All animal testing procedures and care followed the NIH guidelines and were approved by the University of South Florida’s Institutional Animal Care and Use Committee. All methods were carried out in compliance with the ARRIVE guidelines.

### Pharmacological treatment

Mice were assigned pseudo-randomly to groups, and treated with either the casein kinase 1δ/ε inhibitor PF-670462 (10 mg/kg, Cayman Chemical; Ann Arbor, MI) or vehicle (20% (2-hydroxypropyl)-β-cyclodextrin in 25 mM citrate buffer, MilliporeSigma, Oakville, ON) intraperitoneally once daily at ZT10 to correspond to peak CK1 activity in the SCN, beginning 3 days prior to the start of behavioral testing and continuing throughout.

### Behavior

In order to assess the impact of treatment and genotype on learning and memory, we used Fear Conditioning (FC), Morris Water Maze (MWM), Radial Arm Water Maze (RAWM), and Y-maze. We also assessed anxiety and affect using Elevated Plus Maze (EPM), and Porsolt Forced Swim Test (FST). FST was also used as a control for swimming ability. Behavioral testing began on day 4 of treatment with PF-670462 or vehicle and was conducted during the animals’ light cycle, beginning at ZT4 and ending by ZT8. The behavioral battery was scheduled to place more stressful tests at the end of the testing period, and split in to two sets of tests to allow for the use of two different water maze tests. Additionally, each set of tests was split into multiple smaller cohorts of mice to minimize the spread of testing time. Fear conditioning for acute Arc expression was performed on two separate cohorts of mice (Fig. 1c). All behavioral equipment was cleaned with 70% EtOH between mice and trials, a white noise generator was used to reduce outside sounds from disturbing testing, and mice were allowed 30 min to acclimate to the new room before all tests. Entry into any maze segment was scored when all four paws crossed the threshold of the area. Researchers performing behavioral experiments and scoring behavioral data were blinded to the experimental groups.

#### Fear conditioning

Mice were tested in a Plexiglas FC chamber (25 × 25 cm) with metal bar flooring within a sound attenuation chamber and recorded for all sessions. For training, mice were placed in the chamber through a front sliding door and allowed to explore for 3 min with only white noise. Mice were then presented with the conditioned stimulus (CS-90 dB tone) for 30 s, co-terminating with the unconditioned stimulus (US-2 s, 0.50 mA foot shock), 3 and 5 min after the start of the test. 24 h after training, mice were placed back into the same context and allowed to explore for 3 min. One hour after the contextual text, mice were placed in a novel context and presented with 3 min of white noise followed by 3 min of the CS.

#### Morris water maze

Mice were trained in the MWM for 5 days, with 4 trials per day, with at least 30 min to acclimate to the room and between trials. The apparatus consisted of a black pool with a black platform located ~ 2 cm under the surface of the water. Four large visual cues of different colors were mounted on white curtains surrounding the pool. For each trial, the mouse was placed gently in the pool at a different quadrant and allowed 60 s to search for the platform. Mice unable to find the platform after 60 s were guided to it. Mice were left on the platform for 30 s before removal to a recovery cage placed on a heating pad. Training was halted after 5 days as all groups reached an average latency to the platform of < 20 s. Probe trials were conducted at 24 and 72 h after the last training session. The platform was removed, and mice placed in the quadrant opposite its previous position and recorded for 60 s. No differences were found in a visible platform test, ruling out visual deficits in either group.

#### Radial arm water maze

The RAWM consisted of 6 metal arms (20 × 35 cm) in a circular pool, with a center area 40 cm in diameter. Three removable clear Plexiglas platforms served as escape targets for the test, seated in the end of each escape arm under ~ 1 cm of water. Mice were trained for 4 days, each training day consisting of 3 trials per mouse. For trial 1, all 3 platforms were in place, and the mouse was placed in the starting arm, which remained consistent throughout all tests, and was allowed to swim for 2 min or until it “escaped” by finding one of the platforms. The mouse was allowed 30 s to reference the spatial cues from the platform, then was removed, gently dried, and placed in a warmed holding cage for 2 min. For trial 2, the process was repeated with the escape platform used in trial 1 removed, and for trial 3 only a single escape platform remained. Errors were recorded during testing: entries into any arm that never contained a platform were recorded as spatial memory errors and repeated entry into any arm was recorded as a working memory error. An entry was only recorded if all 4 paws entered the arm of the maze. Testing continued until all groups reached the final platform with an average of < 20 s.

#### Y-maze

The spontaneous alternation task was conducted in the Y-maze apparatus, consisting of three arms (38 × 8 cm) connected at 120° angles from each other. Mice were placed in one arm of the maze and allowed to explore for 8 min. Total entries and order of entries was recorded.

#### Elevated plus maze

For EPM testing, mice were placed in the center of a raised apparatus with two opposing open and two opposing closed arms, each (30 × 5 × 15 cm), with a center area (5 × 5 cm), elevated 40 cm off the floor. The apparatus was well lit from above. Mice were placed in the center and recorded for 6 min.

#### Forced swim test

For FST testing, mice were placed in 4 L beakers containing 2.5 L of water at 27 ± 2 °C and monitored. Swimming behavior was recorded for 6 min.

#### Rotarod

Motor coordination testing was performed on a Rotarod apparatus (Ugo Basile) consisting of a 3 cm diameter cylinder machined to provide a grip surface, with 5 lanes. Mice were placed carefully onto the slow-moving rod, and were tested on a ramp of 4–40 rpm over 5 min. Latency to fall was recorded.

#### Grip strength

Forelimb grip strength was tested using a grip strength meter (Ugo Basile) measuring peak force. The mouse was placed over a base plate to grasp a trapeze shaped grasping bar. This was repeated 3 times and the peak force averaged.

#### Hot plate

Pain response was tested using a hot plate apparatus (Ugo Basile), and was essential for ensuring there was no allodynia or hyperalgesia effects that would confound fear conditioning results that were dependent on foot shock. We used a fixed-temperature test, heating the plate surface to 55 °C and placing the mouse in the chamber. The mouse was carefully monitored for hind-leg flick response (or any other indicator of distress), and latency recorded.

#### Sucrose preference

The sucrose preference test was performed overnight during the active (dark) cycle. Mice were singly housed with two sipper bottles, one containing 4% sucrose in water, and one containing water. Food was placed between the bottles. Consumption from each bottle was measured every 3 h, and the bottle position was switched to reduce side-preference confounds. Sucrose preference was calculated as: (sucrose solution consumption)/(total consumption) × 100%.

#### Acute learning paradigm for arc activation

Separate cohorts of mice which had not previously been exposed to the fear conditioning apparatus were used for this experiment. Mice were placed in the fear conditioning chamber and presented with a series of 5 tone-shock pairings, consisting of a 20 s tone (3000 Hz) co-terminating with a foot shock (2 s, 0.50 mA) at 50 s intervals. Mice were placed in separate cages after testing, and tissue was collected exactly 1 h after the end of testing.

Control mice were kept in the home cage until tissue collection.

### Western blot

#### Tissue collection

To generate a snapshot of the expression of circadian and learning and memory related proteins at the time of behavioral testing, mice were euthanized as close as possible to ZT6 the day after a final ZT10 CK1i injection, with isoflurane using the bell jar method. Euthanasia was confirmed by rapid decapitation, and brains were dissected immediately to collect hippocampus and hypothalamus and snap frozen on dry ice. For the Arc expression experiment, euthanasia was performed 1 h after exposure to the learning paradigm (or upon removal from home cage, for the no learning control group) with isoflurane using the bell jar method. Euthanasia was confirmed by rapid decapitation, and brains were dissected to collect right and left amygdala, and dorsal and ventral hippocampus. Dorsal and ventral hippocampus were dissected by cutting at approximately bregma − 4.5 mm. Euthanasia methods used comply with American Veterinary Medical Association (AVMA) humane standards.

#### Western blotting

Western blotting was performed as published previously^58^. Tissue was lysed via sonication in M-PER with protease and phosphatase inhibitors (10 μl/ml, Halt Protease and Phosphatase Inhibitor Cocktail, Thermo Scientific Inc, Rockford, IL, USA) and centrifuged to clear. Supernatant concentrations were detected by BCA Assay (Pierce) and adjusted. Protein was resolved on 9% Tris–glycine polyacrylamide gels under reducing conditions and transferred to nitrocellulose membrane (Bio-rad, Hercules CA). Membranes were blocked for 45 min in 5% milk in tris buffered saline containing 0.1% tween-20 solution (Boston Bioproducts, Ashland, MA). Membranes were incubated overnight at 4 °C in specific antibodies to pERK p44/42, GSK3β, Arg3.1(Arc) (1:1000, Cell Signaling Technology, MA, USA) PER1 (1:1000 Invitrogen, CA, USA), PER2 (1:250 Invitrogen), or BMAL1 (1:1000 Bethyl Laboratories, TX, USA) or for 2 h at RT in antibody specific to β -actin(Sigma-Aldritch, MO, USA) in tris buffered saline with 0.1% tween20 (TBS-T) with 2% milk, washed 3 × 10 min in TBS-T, and incubated for 1 h at room temperature in horseradish peroxidase-conjugated secondary antibody (1:7500, SouthernBiotech, AL, USA), in TBS-T with 2% milk. After washing with TBS-T, bands were visualized with enhanced chemiluminescence (ECL; Thermo scientific) using an image analyzer (Amersham imager 600).

## Data collection and statistical analysis

Behavioral data for MWM and Y-maze were collected using the ANYmaze program. Spontaneous alternations in the Y-maze were calculated as (number of correct triads)/(total number of entries-1), where a correct triad is a sequential entry into each arm without returning to the previously-visited arm. RAWM, FST, EPM, and FC were scored by hand from video recordings by researchers blinded to the experimental conditions. FST was scored by recording the time points during which the mouse was swimming (making forward progress in any direction through the water) and immobile (floating with no motion, minor course correction permitted). Total time immobile and latency to immobility were recorded. EPM was scored by recording the time spent in each of 5 segments of the apparatus: right and left open and closed arms, and center. FC was scored by assessing for freezing behavior of at least 2 s once every 5 s of testing time. Freezing behavior was defined as no movement aside from respiration.

Western blot band intensity was quantified using Image Lab (Bio-Rad). Intensity of bands of interest were normalized to actin band intensity. For Arc expression studies, the VEH + NL group average was set to 1 and all data expressed as fold change from control. For other western blot quantification, VEH group average was set to 1 and all data expressed as fold change from control. Individual outliers falling more than two standard deviations from the group mean were excluded.

Data were analyzed using SPSS version 25 (IBM) and are represented by mean ± SEM, with significance assigned at p < 0.05. Levene’s test or Equality of Variances was used to confirm homogeneity of variance within all data sets.

Training data for RAWM, MWM, and FC were analyzed using a repeated measures ANOVA with the Geisser-Greenhouse correction. Individual outliers falling more than two standard deviations from the group mean were excluded, as well as one mouse with MWM training behavior that fell outside of the confidence intervals for four out of five days of training. Differences in single training days were calculated using unpaired t-tests.

MWM probe trials, characterization studies, and Western blots for GSK3β, PER1, PER2, and BMAL were analyzed using two-tailed, independent samples t-tests. Welch’s t-test was used for contextual FC and pERK expression to correct for significant Levene’s tests for variance within groups.

Spontaneous alternation in the Y-maze was analyzed using a two-tailed, unpaired t-test, and a Pearson’s correlation of spontaneous alternation to distance traveled and number of arm entries was performed to ensure there were no effects of locomotion on the results.

Arc Western blots were analyzed by one-way ANOVA with Tukey post-hoc tests to compare individual differences between groups.

## Supplementary Information

Supplementary Information.

## Data Availability

The datasets generated during and/or analysed during the current study are available from the corresponding author on reasonable request.
