# Supplementary material for: Inhibition of casein kinase 1 δ/ε improves cognitive performance in adult C57BL/6J mice
Source: Sci Rep. 2021 Feb 26;11:4746. doi: 10.1038/s41598-021-83957-9 (PMC7910436; doi:10.1038/s41598-021-83957-9)
Supplement: Supplementary file 1 — Supplementary Information. [file 41598_2021_83957_MOESM1_ESM.docx]

Inhibition of casein kinase 1 δ/ε improves cognitive performance in adult C57BL/6J mice

Heather Mahoney^1^, Emily Peterson^1^, Hannah Justin^1^, David Gonzalez^1^, Christopher Cardona^1^, Korey Stevanovic^2^, John Faulkner^1^, Amara Yunus^3^, Alexandra Portugues^1^, Amy Henriksen^1^, Camden Burns^1^, Cameron McNeill^4^, Joshua Gamsby^1^, Danielle Gulick^1*^

^1^Byrd Alzheimer's Institute, University of South Florida Health, Tampa, FL, USA; Department of Molecular Medicine, Morsani College of Medicine, University of South Florida, Tampa, FL, USA.

^2^National Institute of Environmental Health Sciences, National Institute of Health, Research Triangle Park, NC, USA

^3^University of Florida, College of Pharmacy, Gainesville, FL, USA

^4^USF Health Informatics Institute, University of South Florida Health, Tampa, FL, USA

*Corresponding author: dgulick@usf.edu

**Supplementary Figure 1**: Original, unedited western blot images obtained in .tiff format. Channel and channel+marker .tiff images presented for each, as well as a merged color jpg to view the molecular marker. Channel .tiff images were used for densitometry, and exposure was adjusted to produced main figure images. Red boxes indicate area of original .tiff image that was exposure-corrected and used in the main figure. Blue boxes are displayed on the merged image to more easily visualize which lanes were used.

1. Actin, Hippocampus. Cropped blot appears in main figure 5a.
2. Actin, Hippocampus. Cropped blot appears in main figure 5a.
3. pERK, Hippocampus. Cropped blot appears in main figure 5a.
4. GSK3b, Hippocampus. Cropped blot appears in main figure 5a.
5. PER1, Hippocampus. Cropped blot appears in main figure 5a.
6. BMAL1, Hippocampus, top blot, PER2, hippocampus, bottom blot. Middle two blots unused. Cropped blot appears in main figure 5a.

Lanes are marked as follows:

V: vehicle

C: CK1i

X: controls samples loaded to allow future blots to be combined

1. Arc, Ventral hippocampus, dorsal hippocampus, left amygdala, right amygdala. Cropped blots appear in main figure 6a.
2. Actin, Dorsal hippocampus, ventral hippocampus, left amygdala, right amygdala. Cropped blots appear in main figure 6a.

Lanes are marked as follows:

N: No learning control

V: Vehicle + learning

C: CK1i + learning


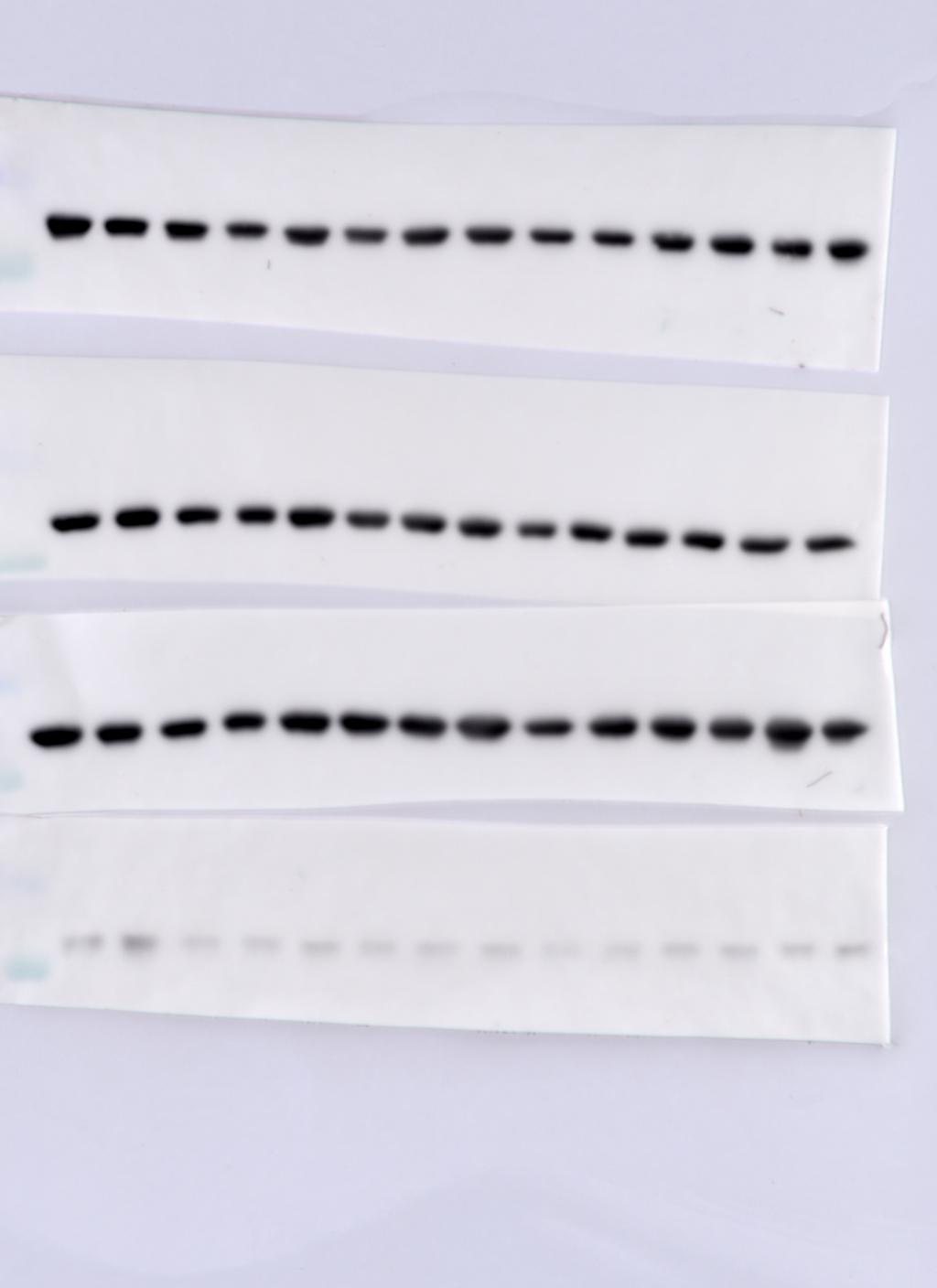

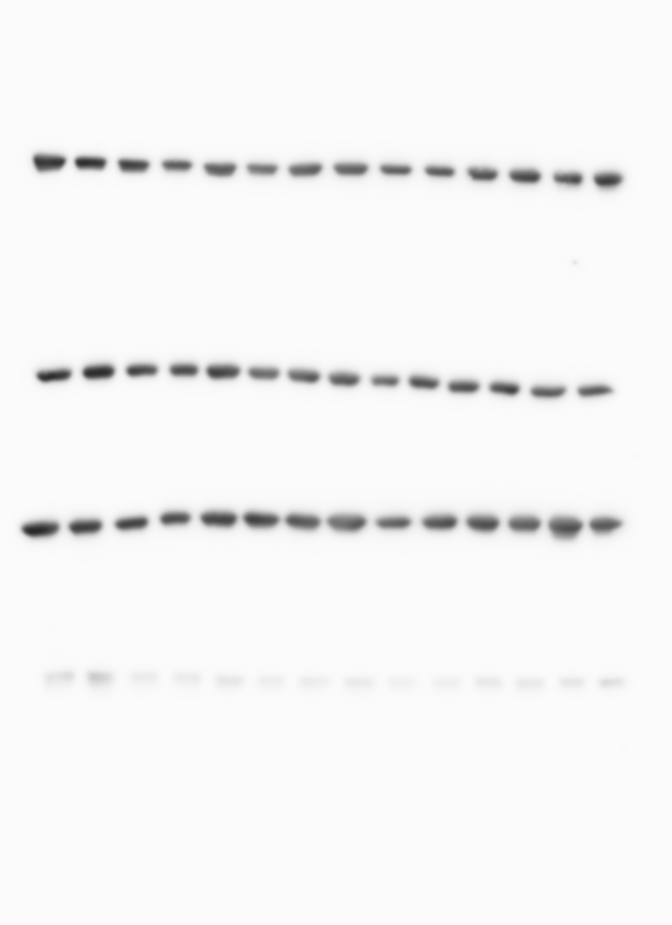


V V V V V V C C C C C C X X

V V V V V V C C C C C C X X

50kda

A

37

V V V V V V C C C C C C X X

V V V V V V C C C C C C X X

50kda


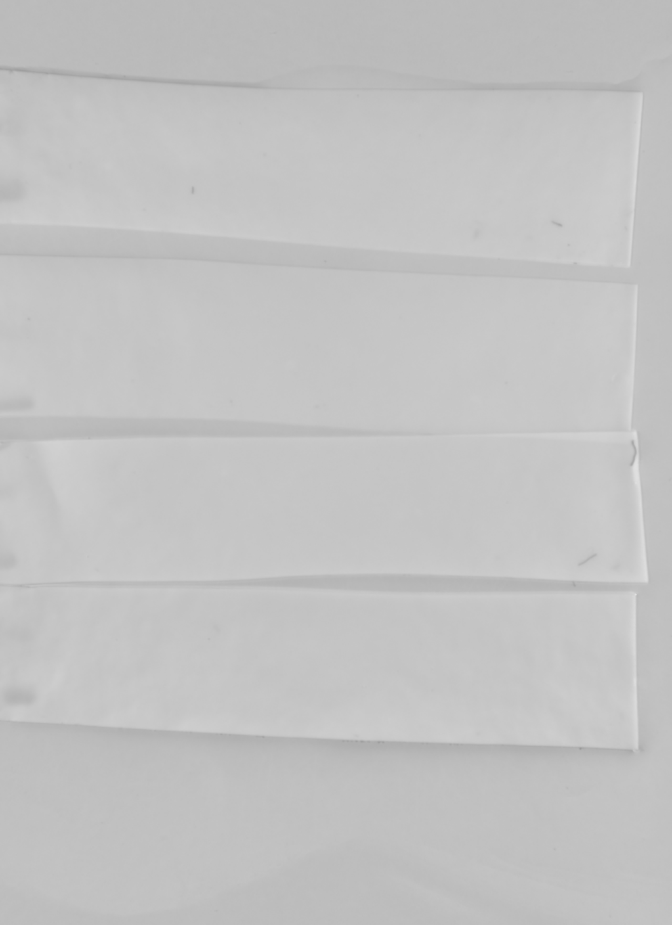


37

50kda

37

V V V V V V C C C C C C X X

V V V V V V C C C C C C X X

V V V V V V C C C C C C X X

V V V V V V C C C C C C X X

V V V V V V C C C C C C X X

1. Actin, Hippocampus, Cropped blot appears in main figure 5a.

V: vehicle

C: CK1i

X: controls samples loaded to allow future blots to be combined

Red boxes indicate area of original .tiff image that was exposure-corrected and used in the main figure.

Blue boxes are displayed on the merged image to more easily visualize which lanes were used.

B


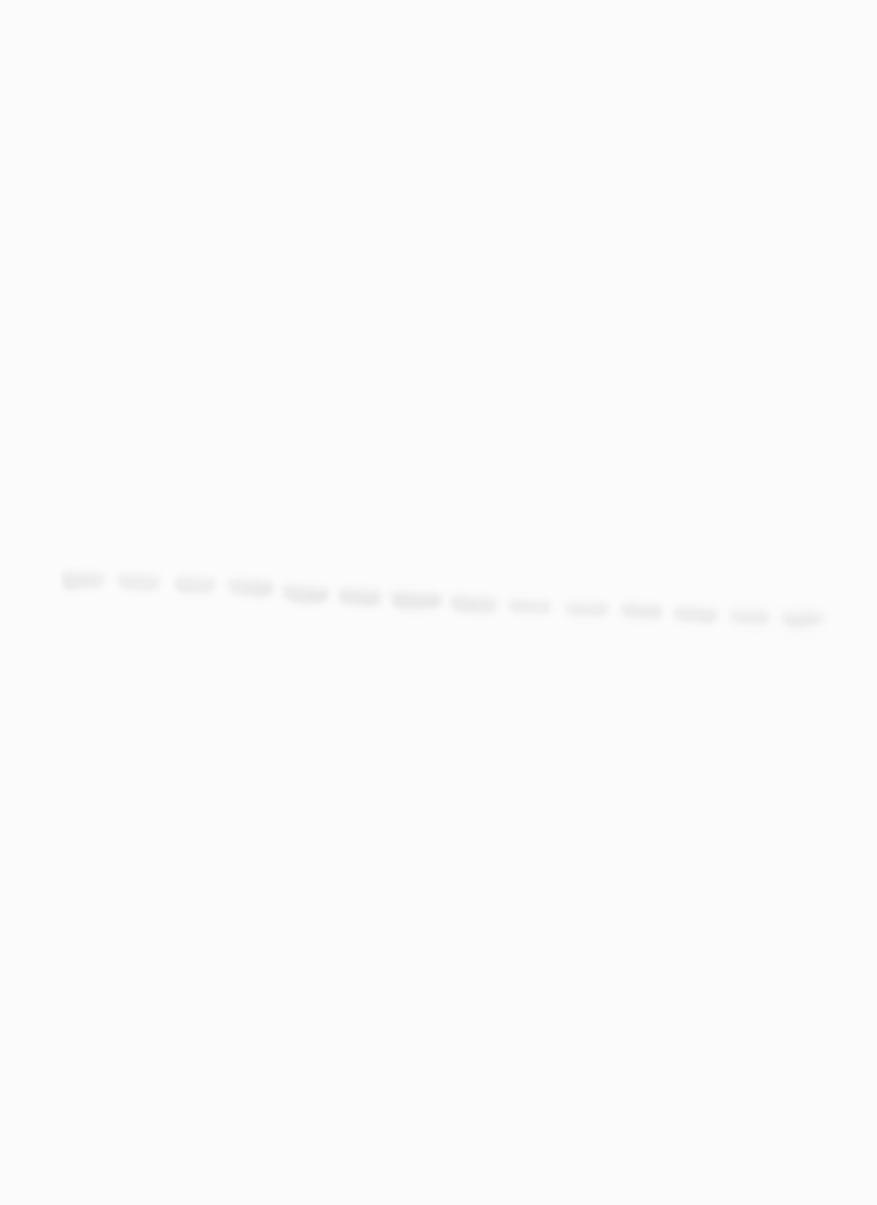


V V V V V V C C C C C C X X

1. Actin, Hippocampus. Cropped blot appears in main figure 5a.

V: vehicle

C: CK1i

X: controls samples loaded to allow future blots to be combined

Red boxes indicate area of original .tiff image that was exposure-corrected and used in the main figure.

Blue boxes are displayed on the merged image to more easily visualize which lanes were used.

50kda

37


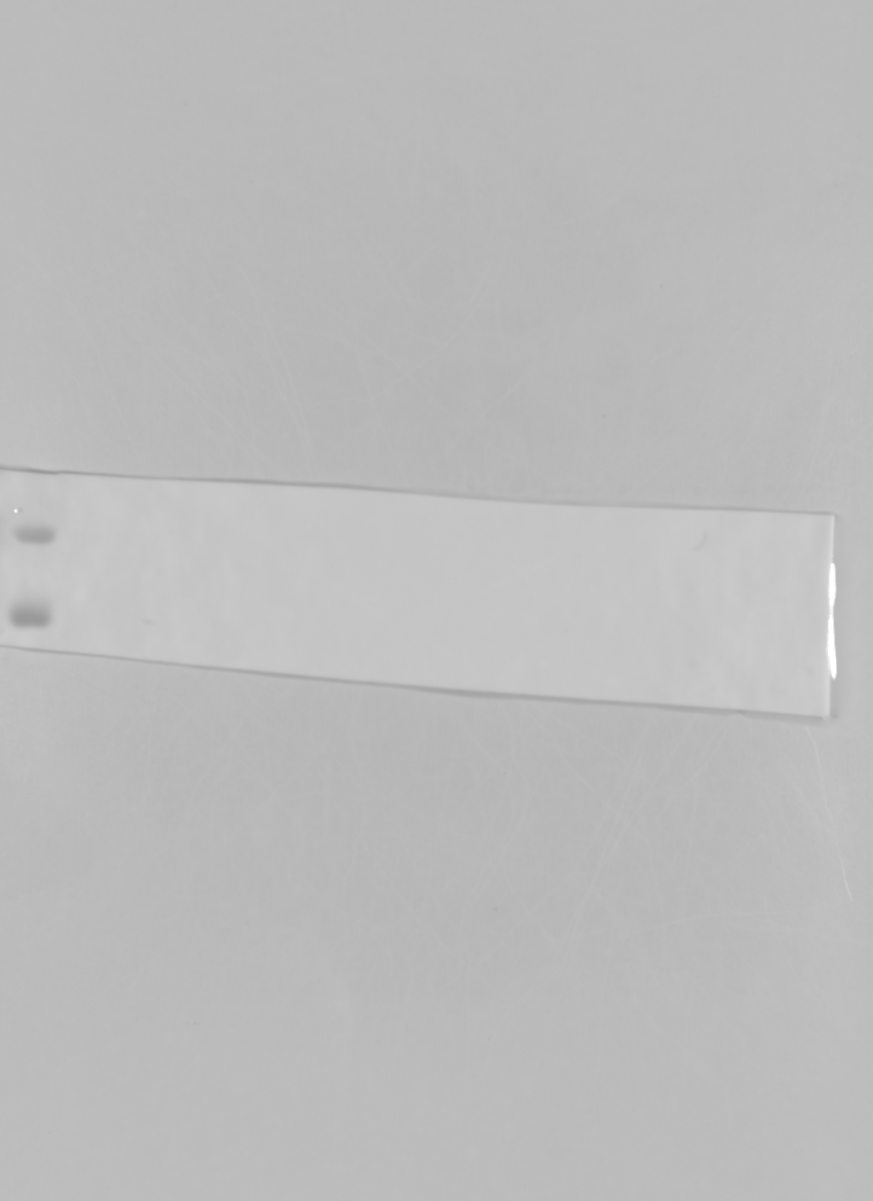


V V V V V V C C C C C C X X


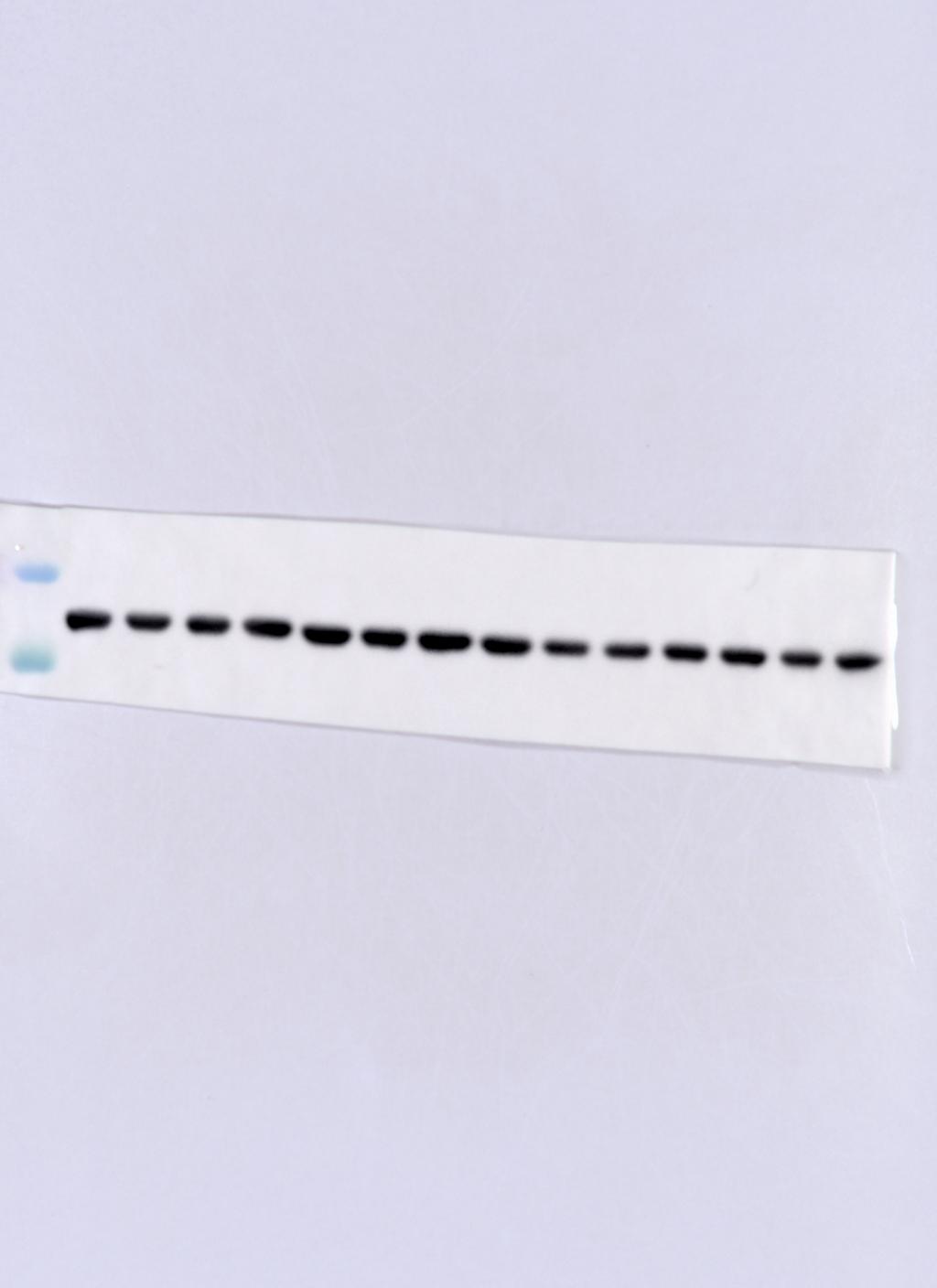


V V V V V V C C C C C C X X

C


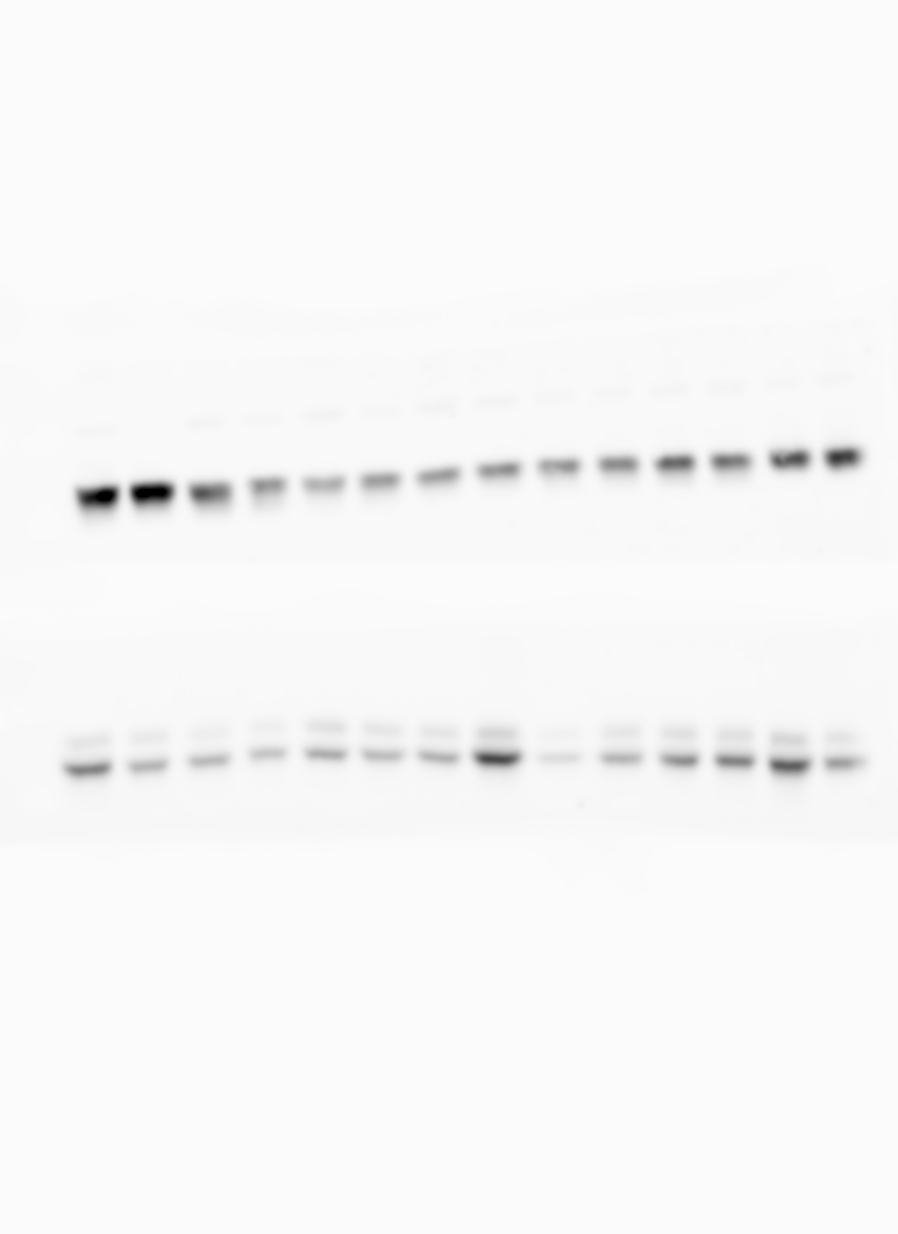

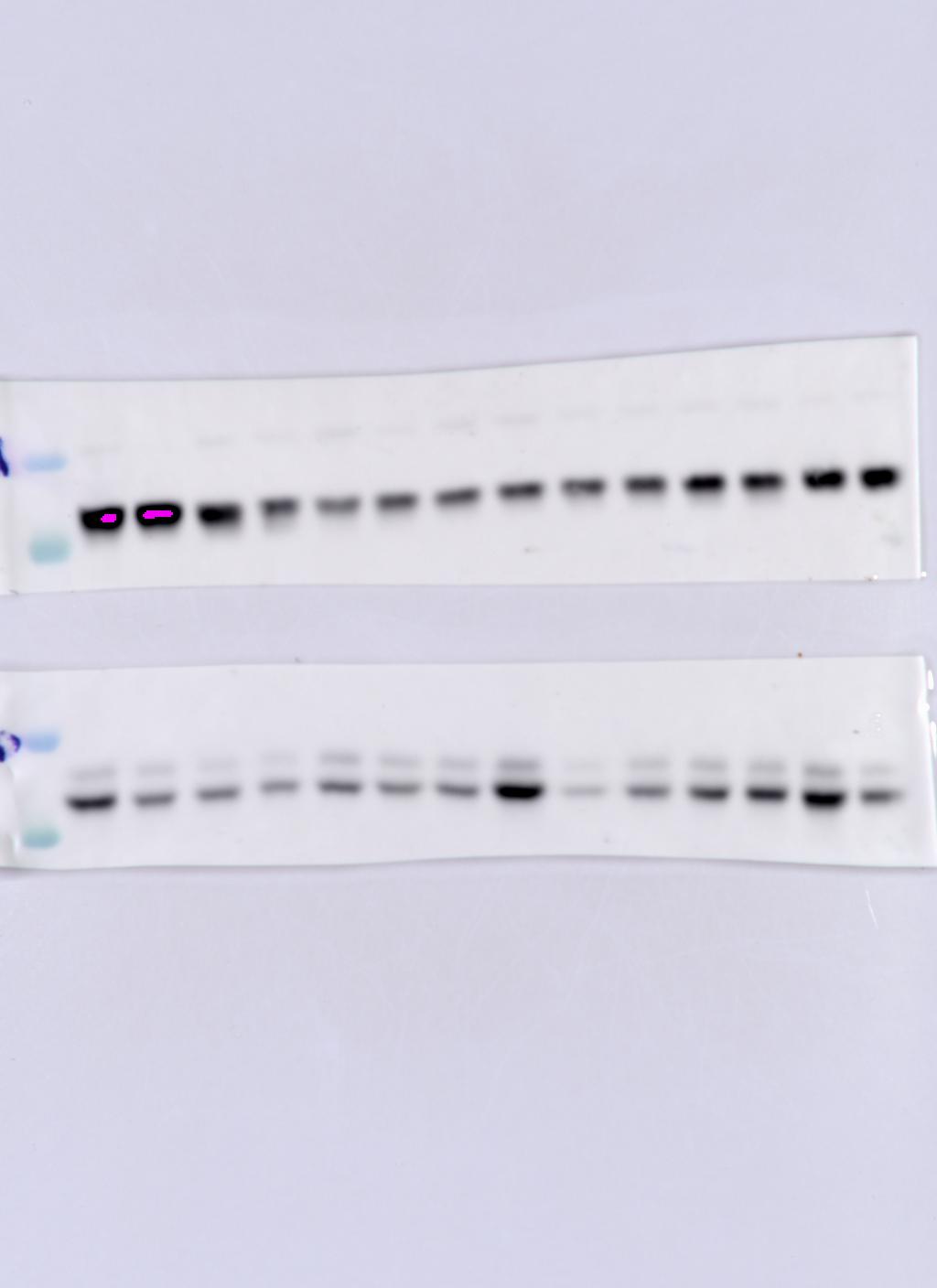

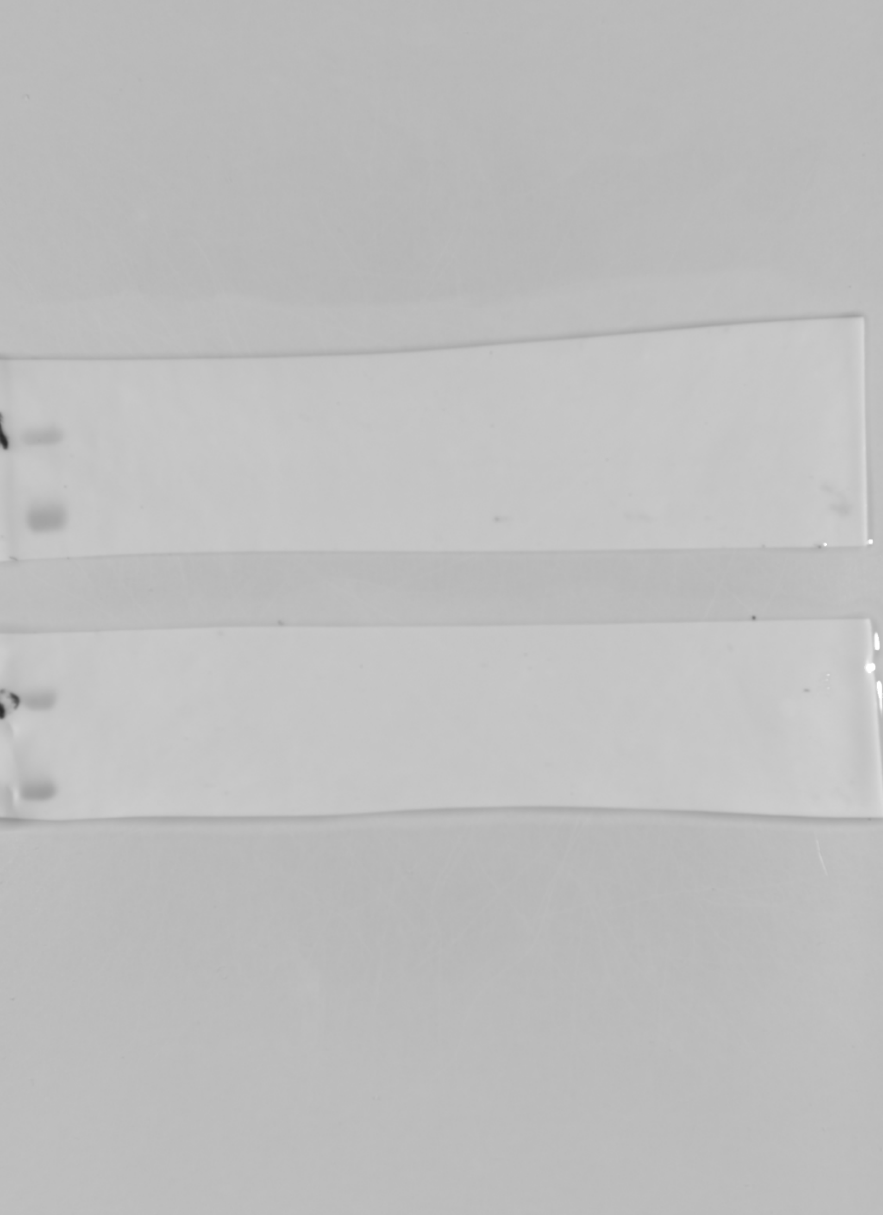


50kda

37

V V V V V V C C C C C C X X

V V V V V V C C C C C C X X

V V V V V V C C C C C C X X

1. pERK, Hippocampus, bottom blot. Top blot unused.

Cropped blot appears in main figure 5a.

V: vehicle

C: CK1i

X: controls samples loaded to allow future blots to be combined

Red boxes indicate area of original .tiff image that was exposure-corrected and used in the main figure.

Blue boxes are displayed on the merged image to more easily visualize which lanes were used.


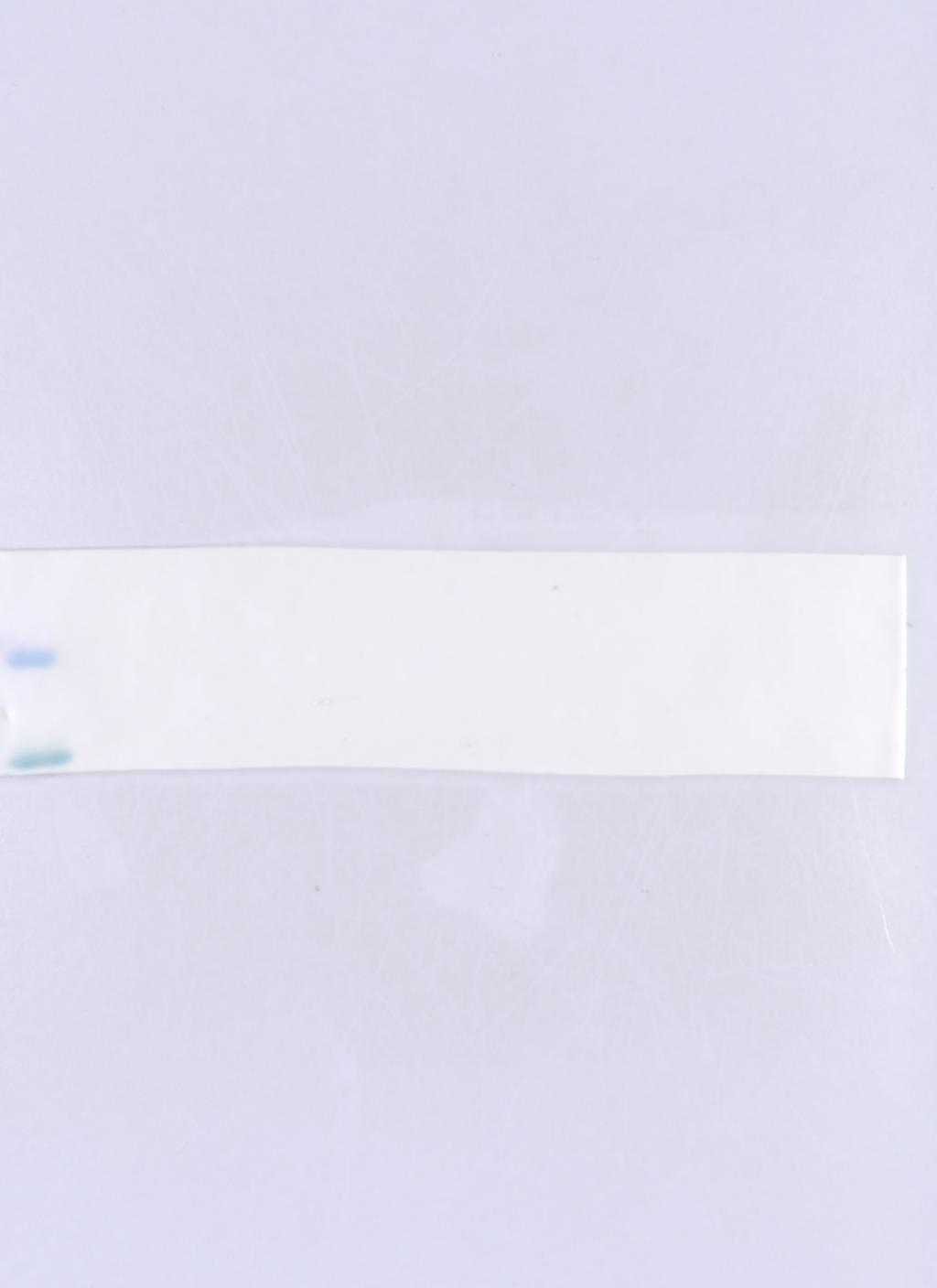

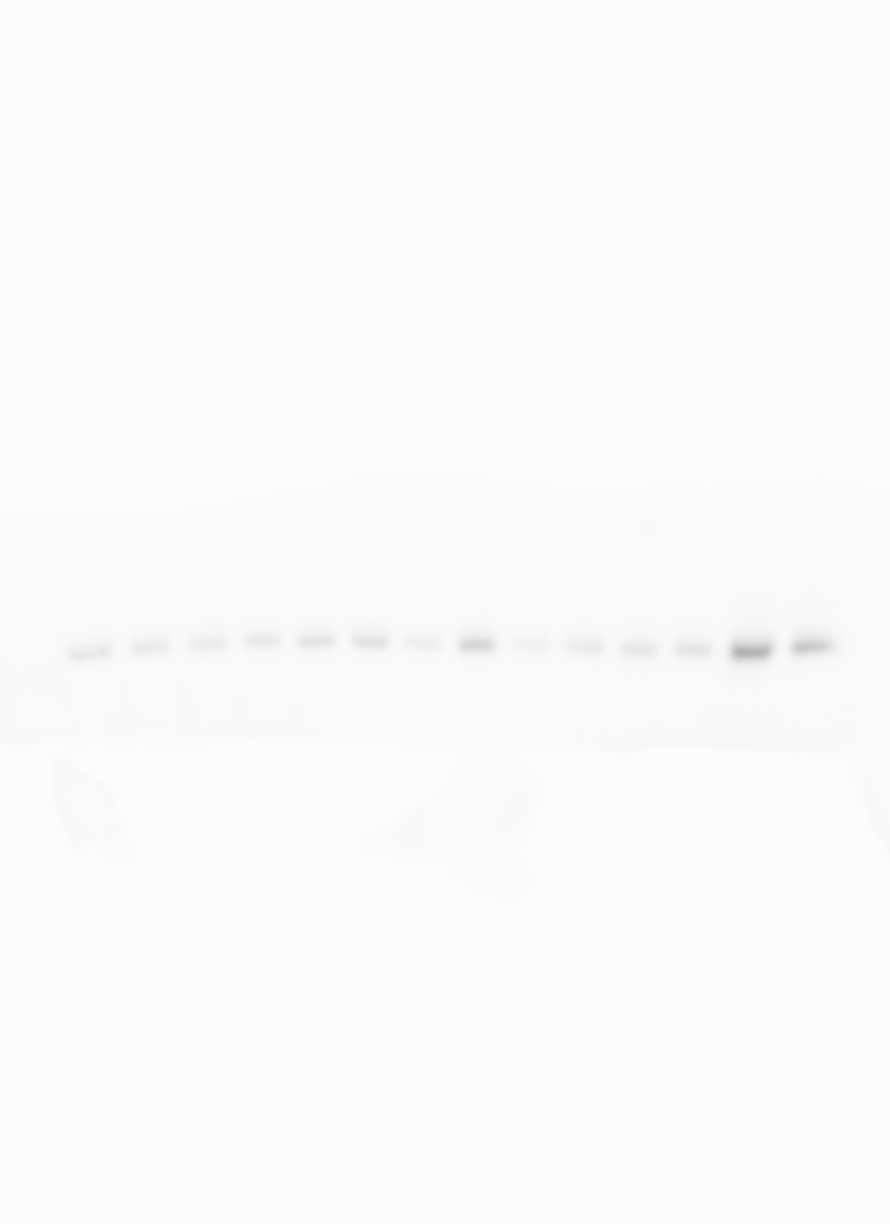


D

V V V V V V C C C C C C X X

V V V V V V C C C C C C X X

50kda

37


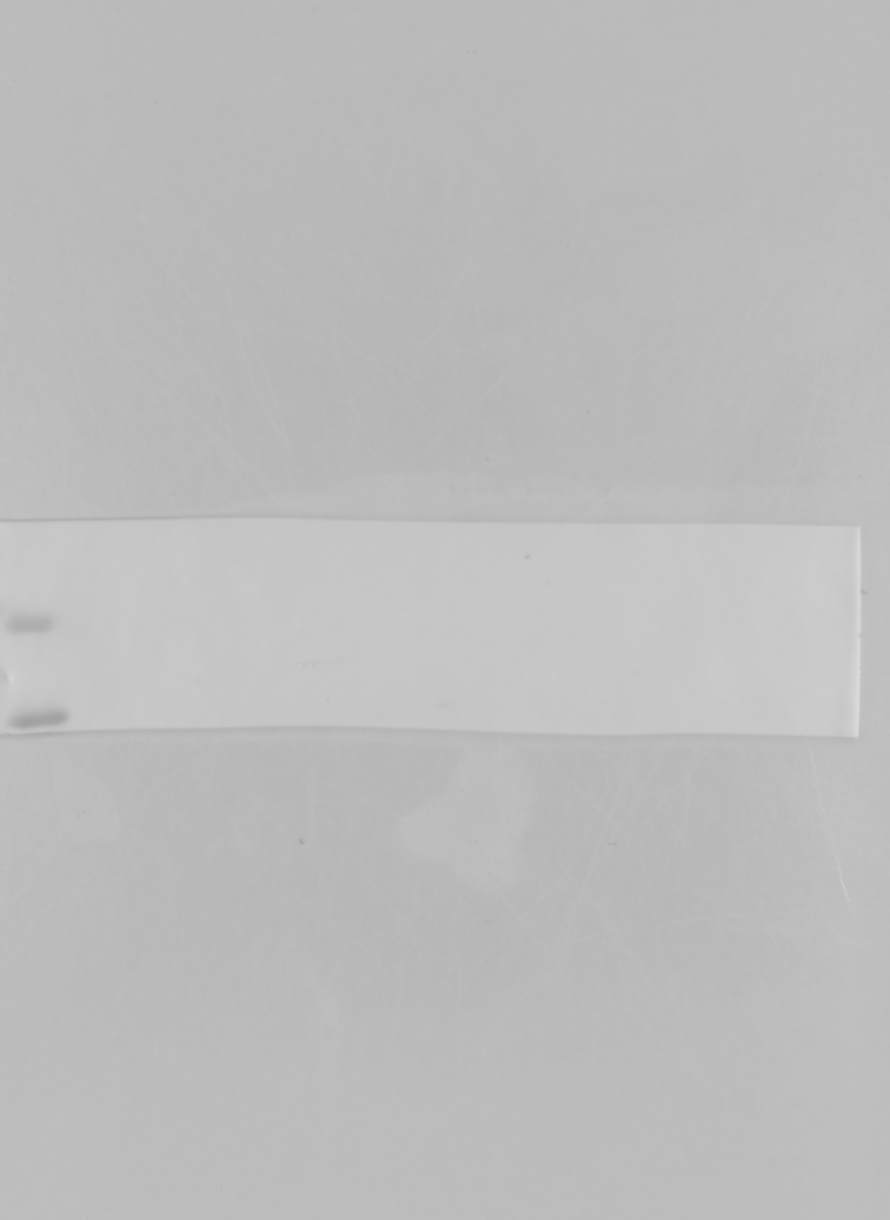


V V V V V V C C C C C C X X

1. GSK3b, Hippocampus. Cropped blot appears in main figure 5a.

V: vehicle

C: CK1i

X: controls samples loaded to allow future blots to be combined

Red boxes indicate area of original .tiff image that was exposure-corrected and used in the main figure.

Blue boxes are displayed on the merged image to more easily visualize which lanes were used.

E


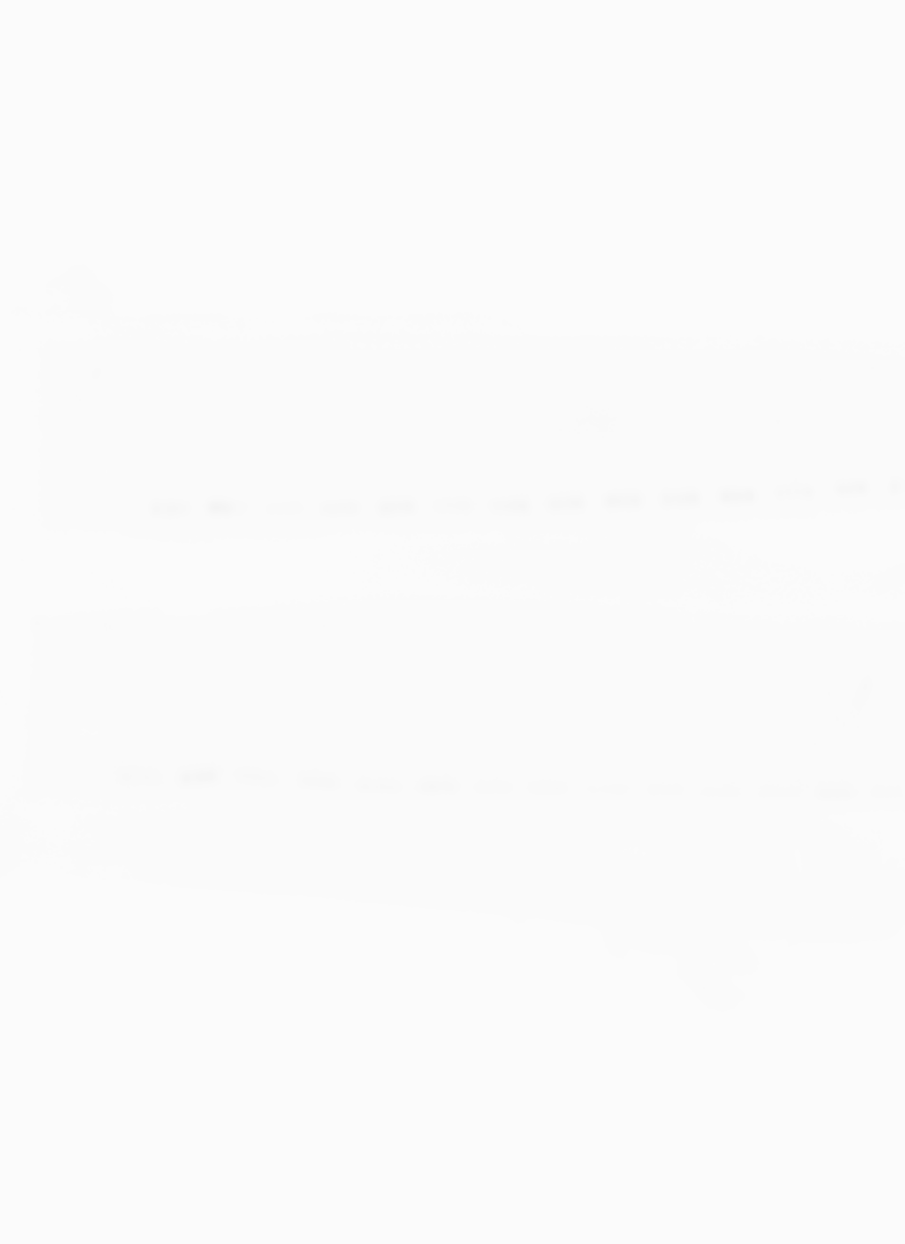


V V V V V V C C C C C C X X


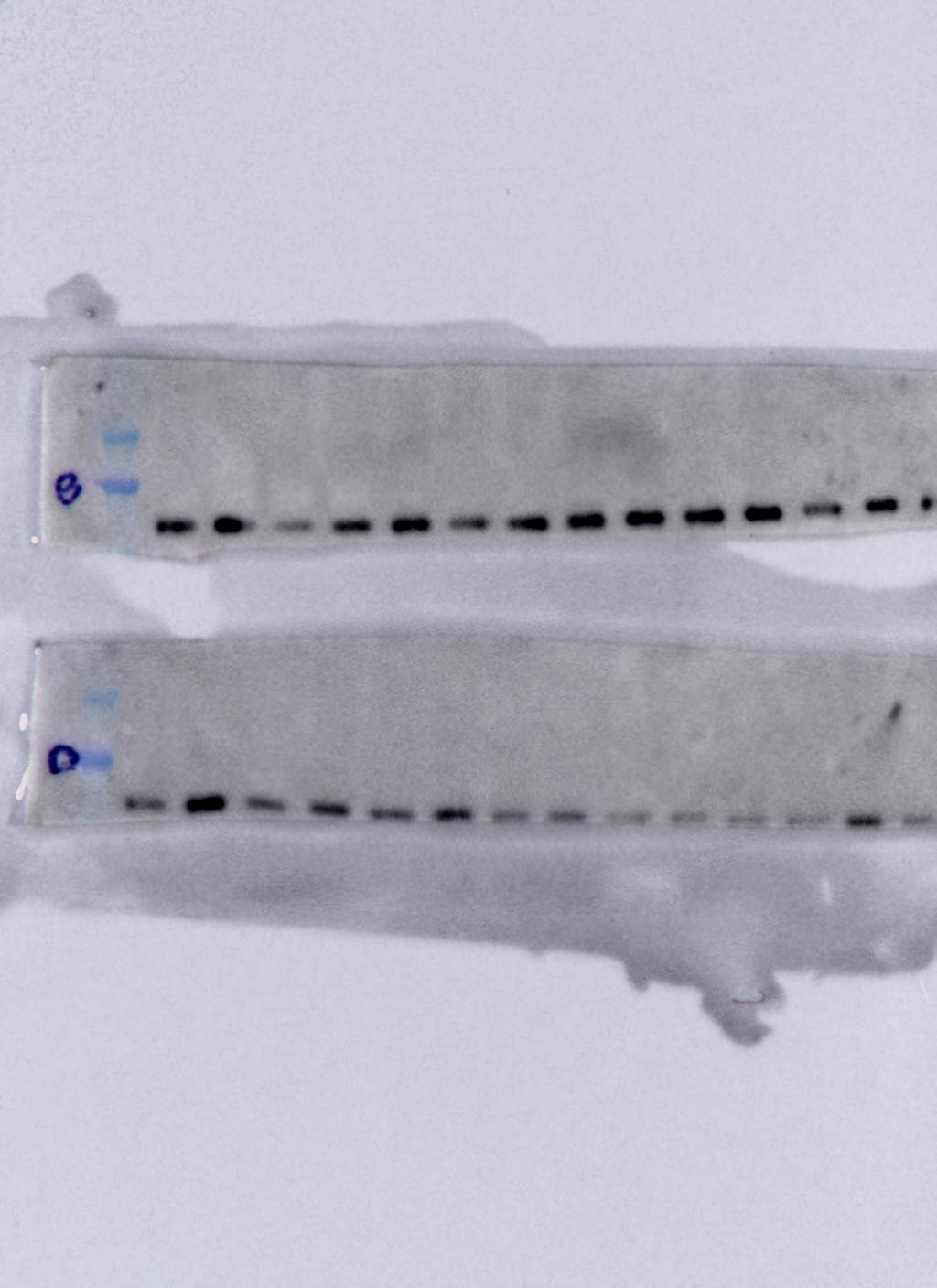


250kda

V V V V V V C C C C C C X X

15

100


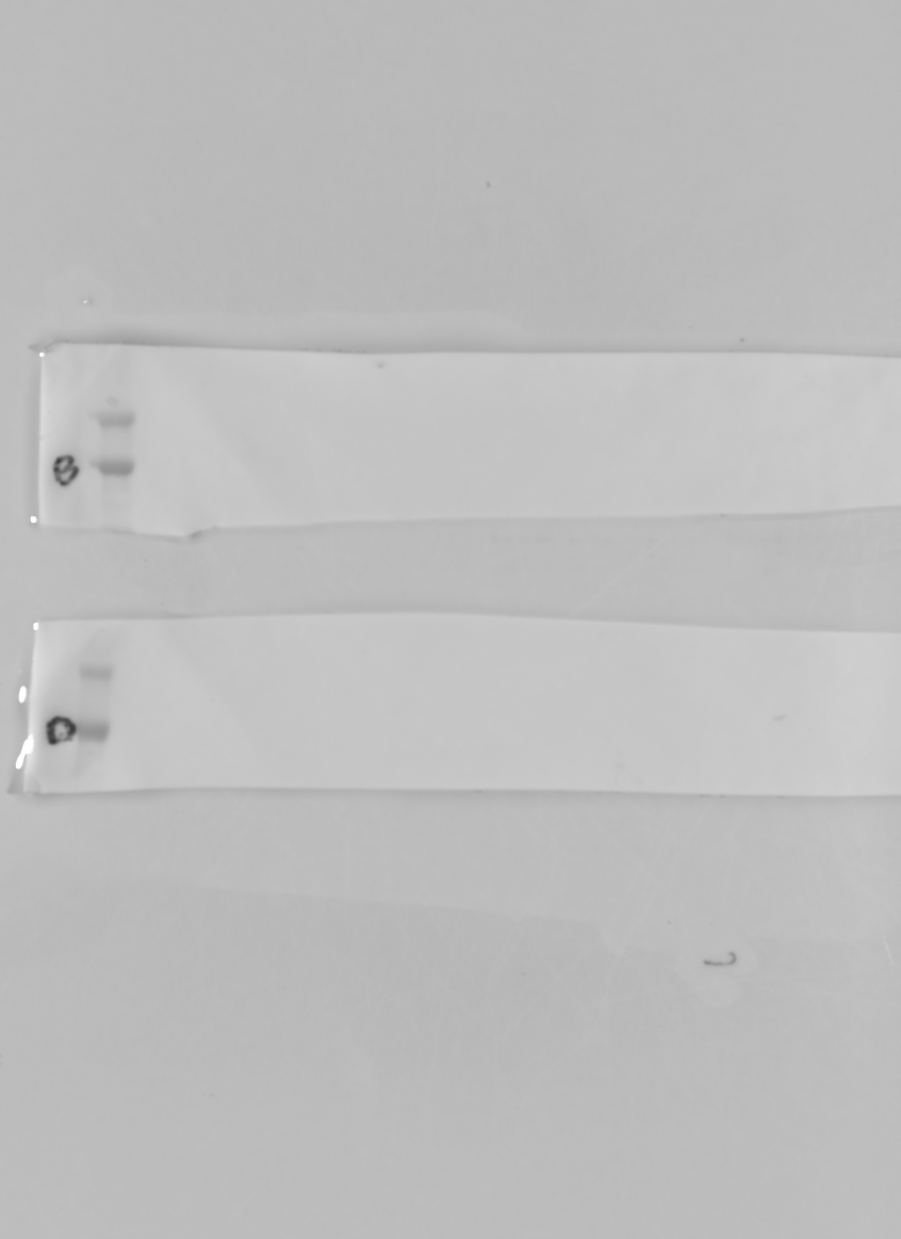


V V V V V V C C C C C C X X

1. PER1, Hippocampus. Cropped blot appears in main figure 5a.

V: vehicle

C: CK1i

X: controls samples loaded to allow future blots to be combined

Red boxes indicate area of original .tiff image that was exposure-corrected and used in the main figure.

Blue boxes are displayed on the merged image to more easily visualize which lanes were used.


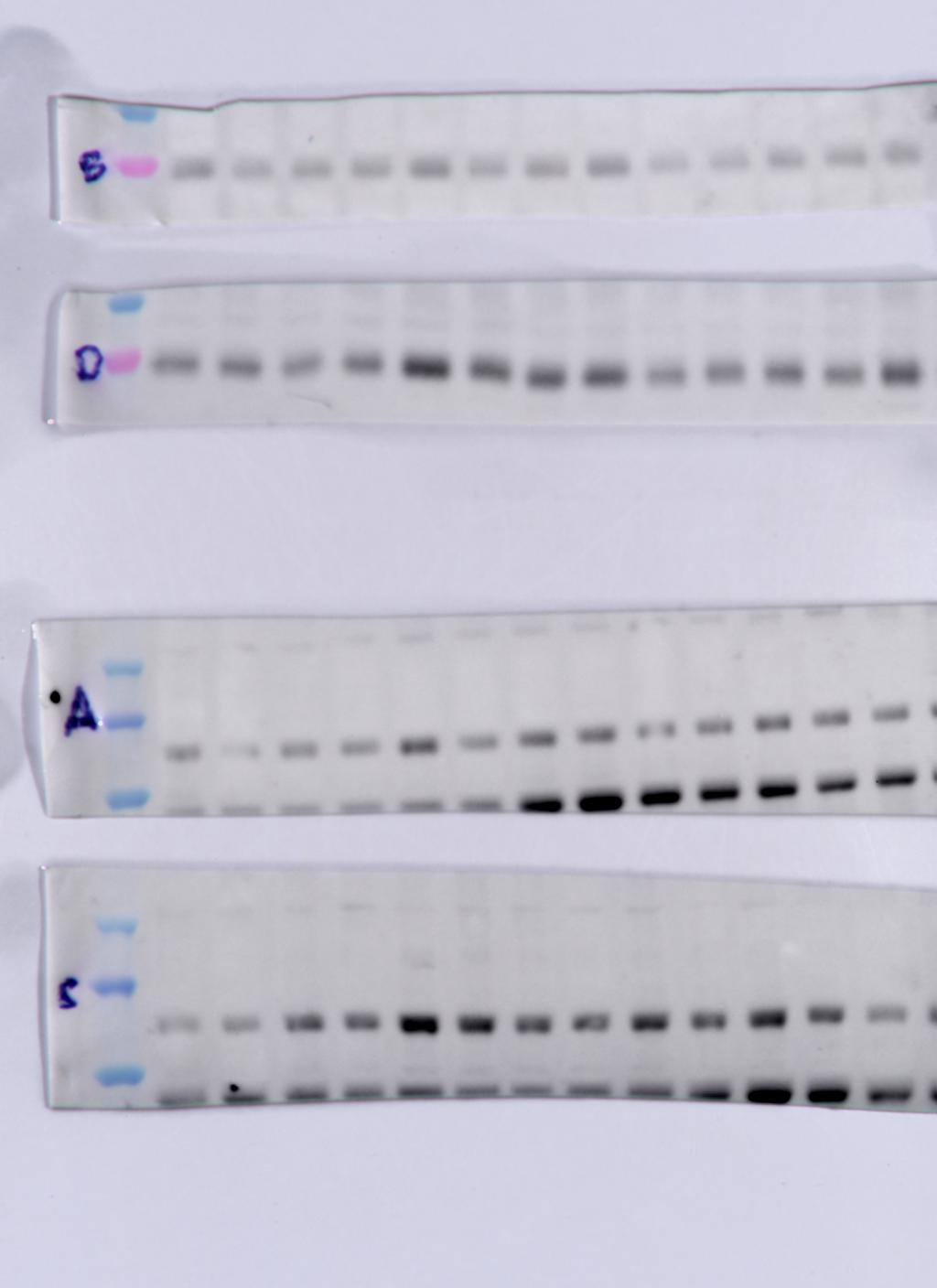

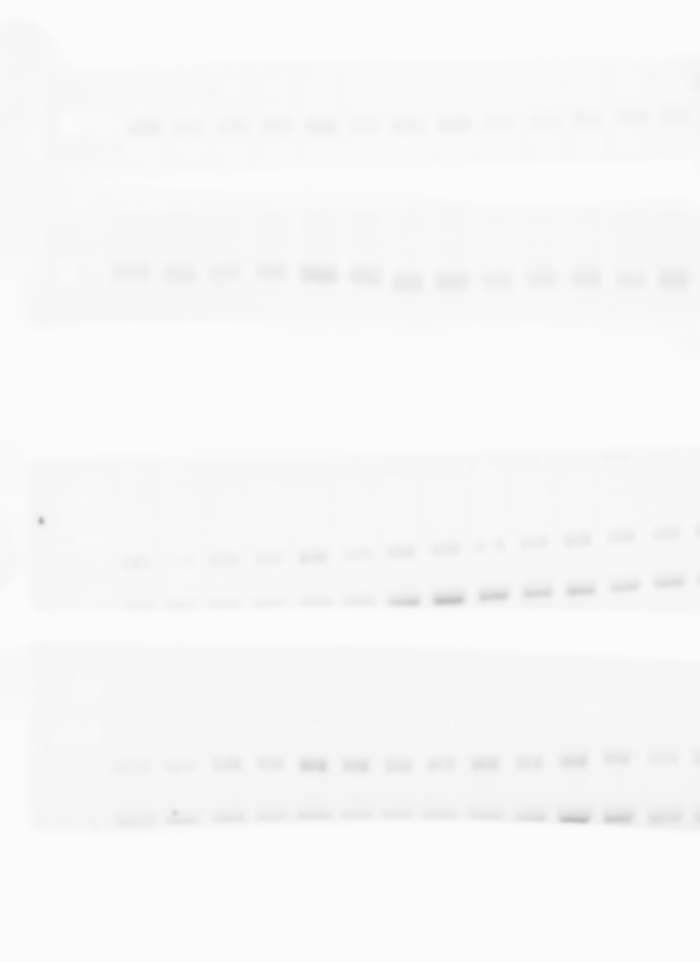

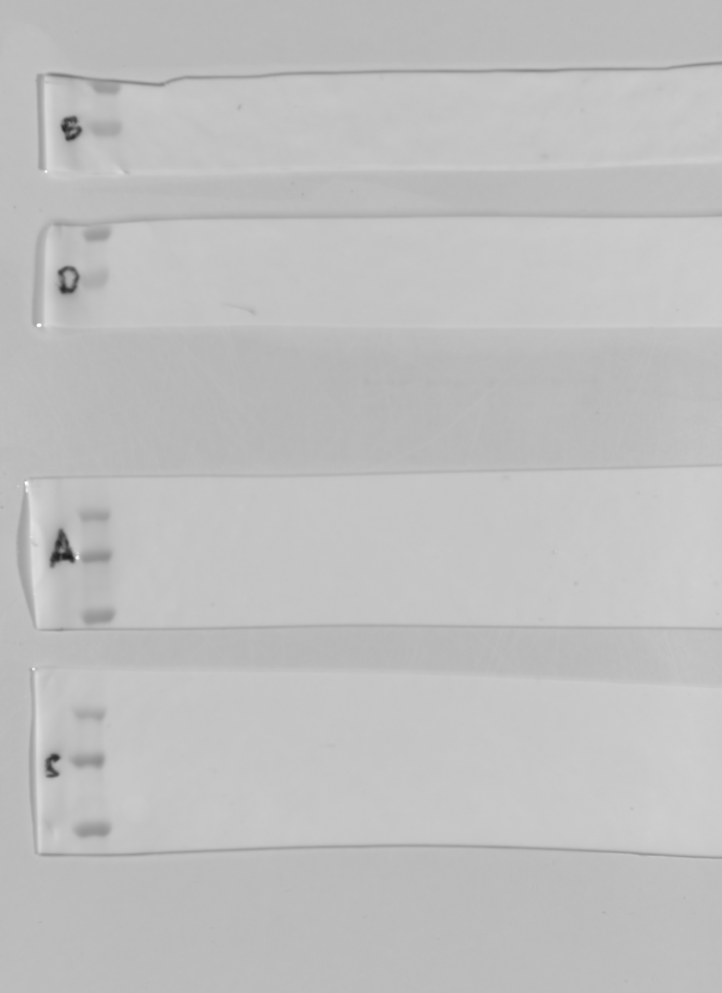


100

150

250kda

75

100kda

F

V V V V V V C C C C X X X X

V V V V V V C C C C C C X X

V V V V V V C C C C C C X X

V V V V V V C C C C C C X X

V V V V V V C C C C C C X X

1. BMAL1, Hippocampus, top blot, PER2, hippocampus, bottom blot. Middle two blots unused. Cropped blots appear in main figure 5a.

V: vehicle

C: CK1i

X: controls samples loaded to allow future blots to be combined

Red boxes indicate area of original .tiff image that was exposure-corrected and used in the main figure.

Blue boxes are displayed on the merged image to more easily visualize which lanes were used.

V V V V V V C C C C X X X X


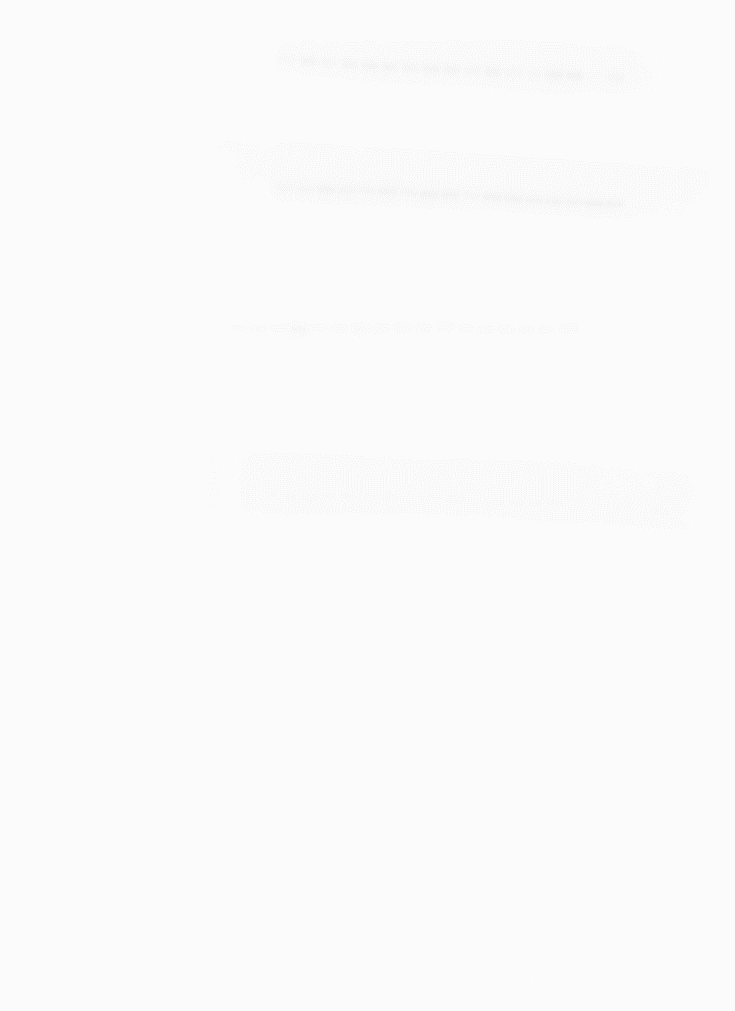


G


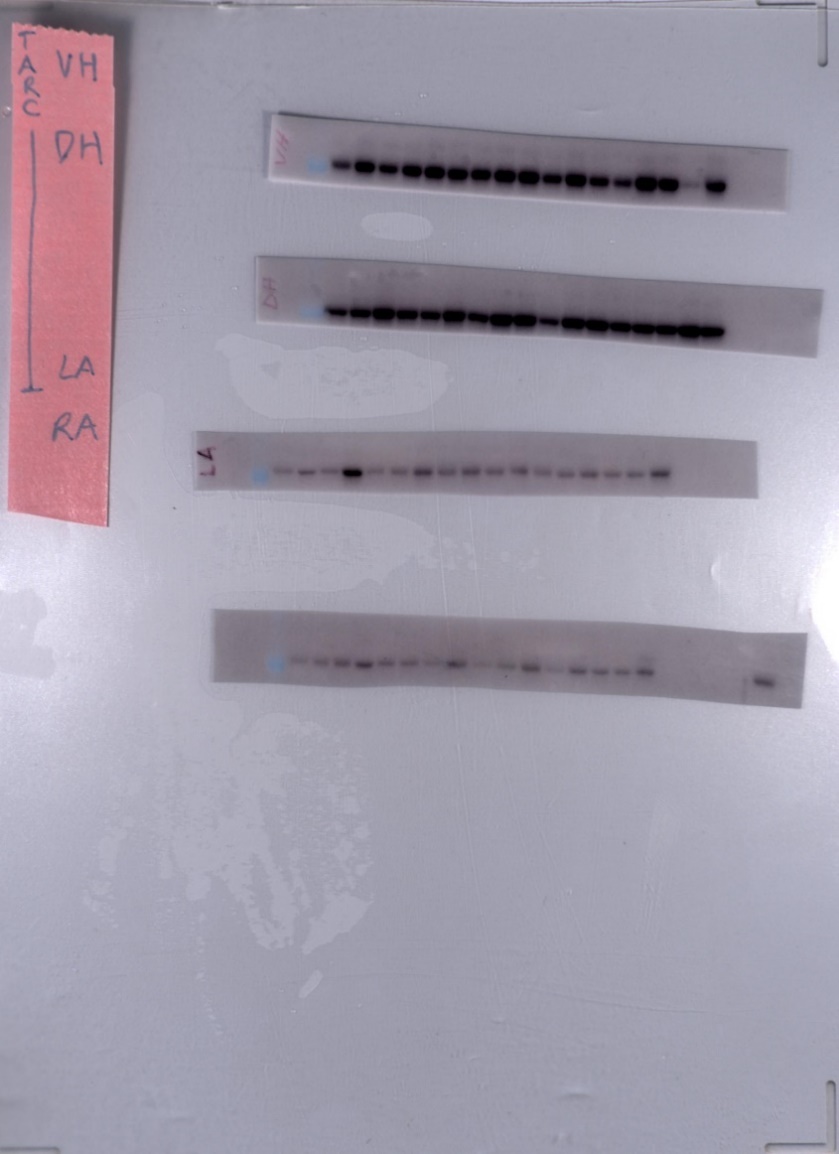


N V C N V C N V C N V C N V C V C

N V C N V C N V C N V C N V C V C

N V C N V C V N V C N V C C N V C

N V C N V C N C N V C N V C V C V

50kda

N V C N V C N V C N V C N V C V C

N V C N V C N V C N V C N V C V C

N V C N V C V N V C N V C C N V C

N V C N V C N C N V C N V C V C V

50kda

50kda

+

1. Arc, Top to bottom: Ventral hippocampus, dorsal hippocampus, left amygdala, right amygdala. Cropped blots appear in main figure 6a.

N: No learning control

V: Vehicle + learning

C: CK1i + learning

Top left: Red boxes indicate area of original .tiff image that was exposure-corrected and used in the main figure.

Top right: Blue boxes are displayed on the merged image to more easily visualize which lanes were used.

Bottom left: Top left .tiff channel image enhanced in Biorad Image Lab 6.1 to make blots are visible to the eye

Bottom right: marker-only image


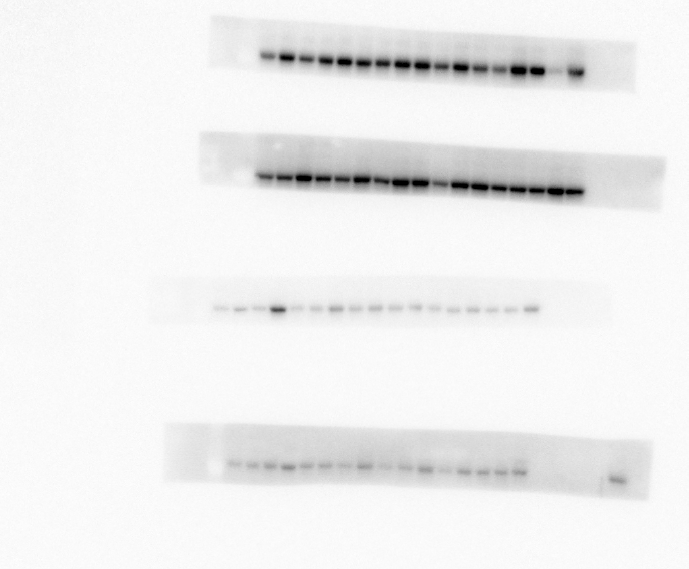


N V C N V C N V C N V C N V C V C

N V C N V C N V C N V C N V C V C

N V C N V C V N V C N V C C N V C

N V C N V C N C N V C N V C V C V


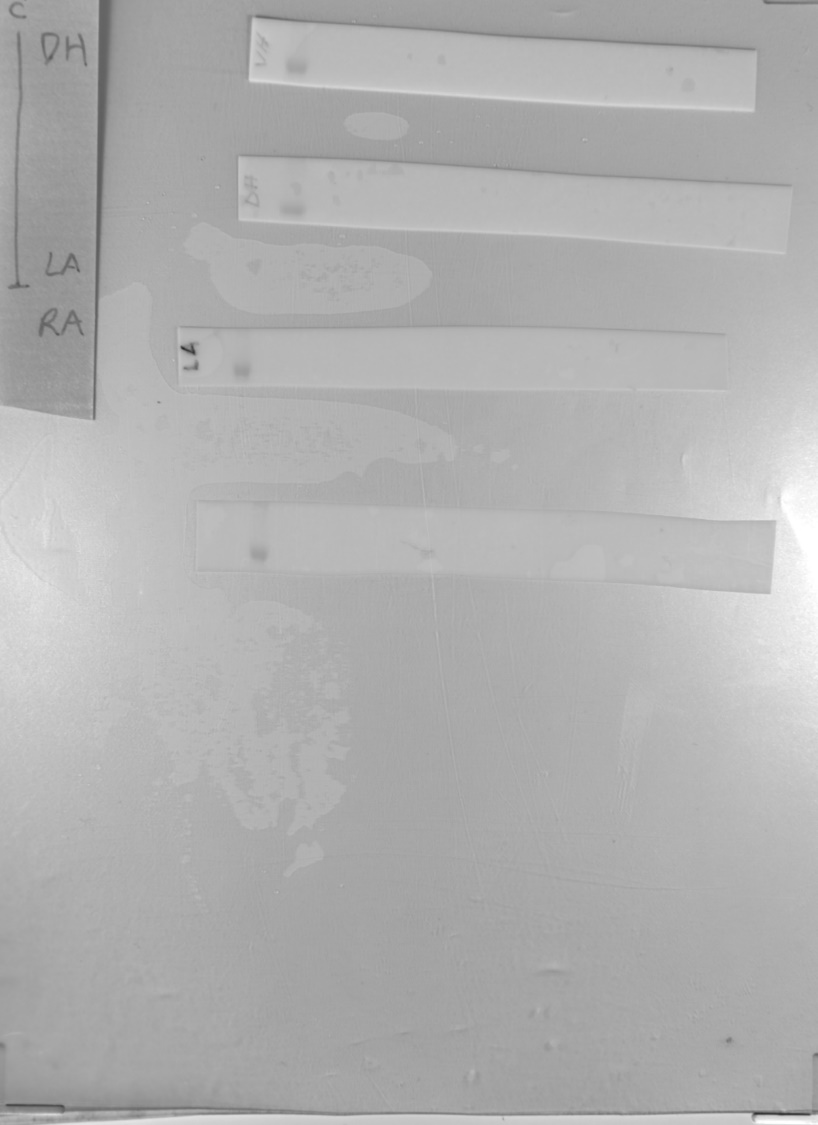


N V C N V C N V C N V C N V C V C

N V C N V C N V C N V C N V C V C

N V C N V C V N V C N V C C N V C

N V C N V C N C N V C N V C V C V

50kda


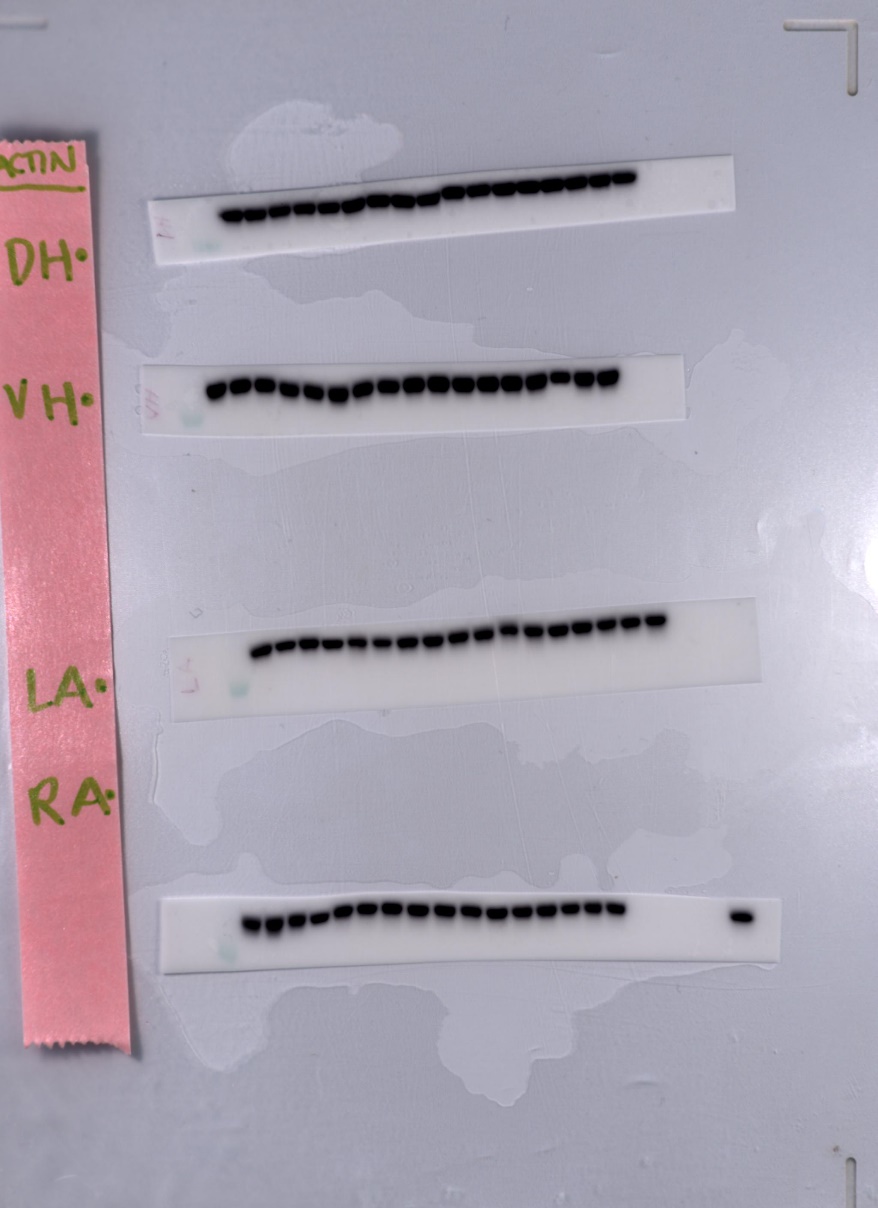

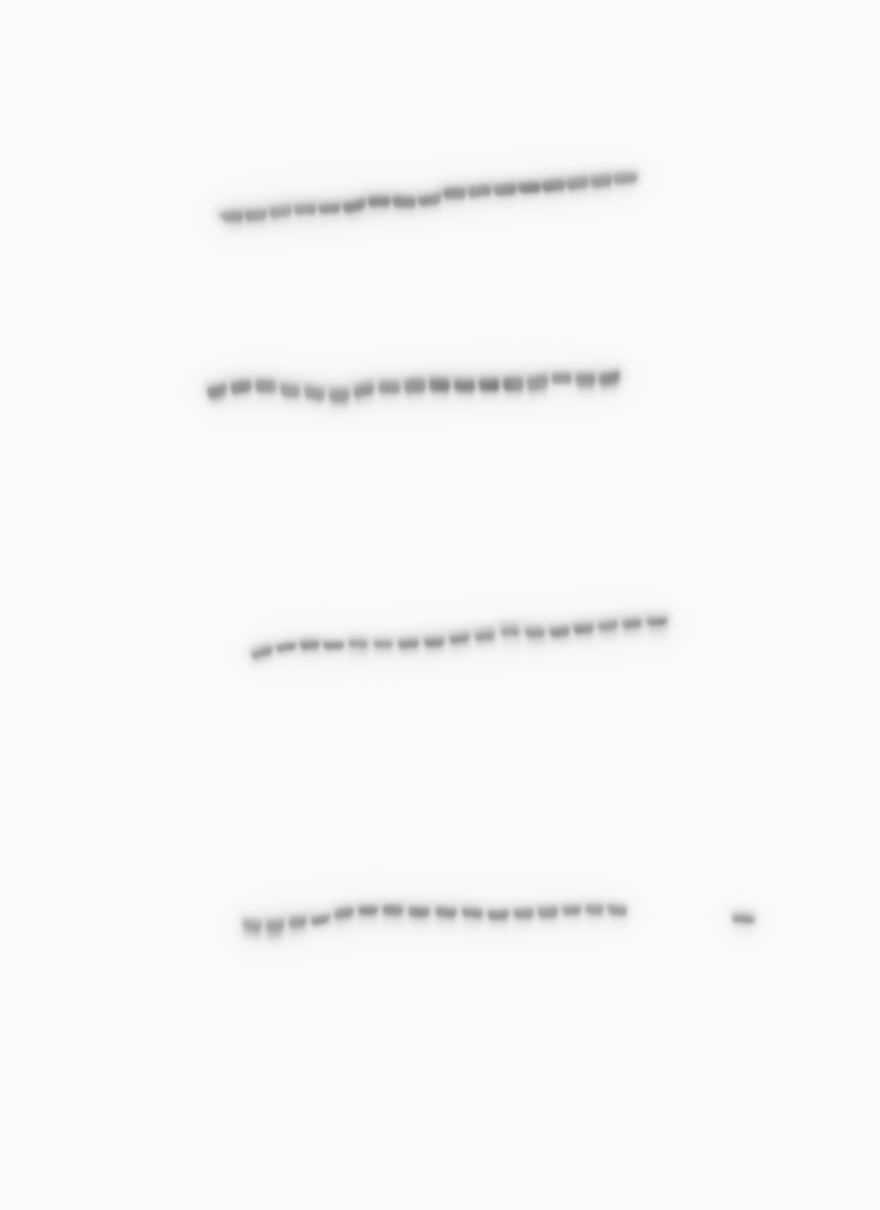


H

N V C N V C N V C N V C N V C V C

N V C N V C N V C N V C N V C V C

1. Actin, Dorsal hippocampus, ventral hippocampus, left amygdala, right amygdala. Cropped blots appear in main figure 6a.

N: No learning control, V: Vehicle + learning, C: CK1i + learning

Red boxes indicate area of original .tiff image that was exposure-corrected and used in the main figure.

Blue boxes are displayed on the merged image to more easily visualize which lanes were used.


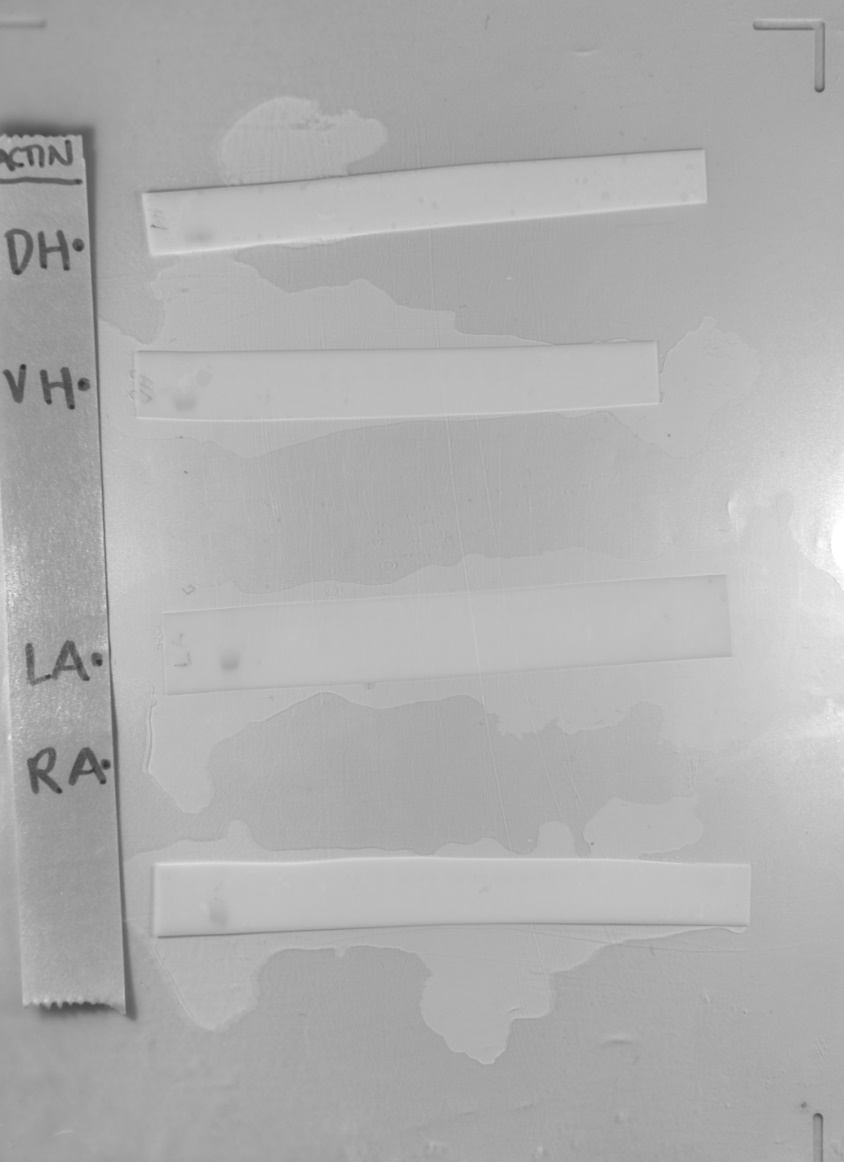


N V C N V C V N V C N V C C N V C

N V C N V C N V C N V C N V C V C

N V C N V C N V C N V C N V C V C

N V C N V C N C N V C N V C V C V

N V C N V C V N V C N V C C N V C

N V C N V C N V C N V C N V C V C

N V C N V C N C N V C N V C V C V

N V C N V C V N V C N V C C N V C

N V C N V C N V C N V C N V C V C

N V C N V C N C N V C N V C V C V

37kda

37kda

37kda

37kda
